# Activity of cefepime/taniborbactam and comparators against whole genome sequenced ertapenem-non-susceptible Enterobacterales clinical isolates: CANWARD 2007–19

**DOI:** 10.1093/jacamr/dlab197

**Published:** 2022-02-07

**Authors:** Alyssa R. Golden, Melanie R. Baxter, James A. Karlowsky, Laura Mataseje, Michael R. Mulvey, Andrew Walkty, Denice Bay, Frank Schweizer, Philippe R. S. Lagace-Wiens, Heather J. Adam, George G. Zhanel

**Affiliations:** 1 Department of Medical Microbiology and Infectious Diseases, Max Rady College of Medicine, University of Manitoba, Room 543-745 Bannatyne Avenue, Winnipeg, Manitoba, R3E 0J9, Canada; 2 Clinical Microbiology, Health Sciences Centre/Diagnostic Services, Shared Health, MS673-820 Sherbrook Street, Winnipeg, Manitoba, R3A 1R9, Canada; 3 National Microbiology Laboratory, Public Health Agency of Canada, 1015 Arlington Street, Winnipeg, Manitoba, R3E 3R2, Canada; 4 Department of Chemistry, Faculty of Science, University of Manitoba, Room 448 Parker Bldg, 144 Dysart Rd, Winnipeg, Manitoba, R3 T 2N2, Canada

## Abstract

**Objectives:**

This study assessed *in vitro* activities of cefepime/taniborbactam and comparator antimicrobial agents against ertapenem-non-susceptible Enterobacterales (ENSE) clinical isolates collected from the CANWARD study 2007–19, and associations between MIC and various mechanisms of β-lactam resistance identified using WGS.

**Methods:**

A total of 179 ENSE (MIC ≥ 1 mg/L) isolates underwent susceptibility testing using reference CLSI broth microdilution. WGS was performed using the Illumina NextSeq platform. Carbapenemases, ESBLs and other β-lactamases were identified using ResFinder 4.0. Alterations in *ompC*/*F* and *ftsI* (PBP3) were identified by comparing extracted sequences to the appropriate NCBI reference gene. Porin alterations were analysed with Provean v1.1.3. Specific alterations of interest in PBP3 included a YRIN or YRIK insertion after P333.

**Results:**

Cefepime/taniborbactam was highly active (MIC_50_/MIC_90_, 0.5/2 mg/L; 177/179 isolates inhibited at ≤ 8 mg/L) against ENSE with various antimicrobial resistance phenotypes. Thirteen (7.3%) of the 179 ENSE isolates demonstrated cefepime/taniborbactam MIC values ≥ 4 mg/L and possessed combinations of β-lactam resistance mechanisms, including a carbapenemase and/or ESBL and/or other β-lactamase genes, as well as alterations in OmpC and/or OmpF and/or PBP3. Of the two *Escherichia coli* isolates that demonstrated a cefepime/taniborbactam MIC of 32 mg/L, one possessed NDM-5, OXA-181 and TEM-1B, an OmpC alteration and P333_Y334insYRIN in PBP3, while the second contained CTX-M-71, a truncated OmpF and a large alteration in OmpC (F182_R195delinsMTTNGRDDVFE).

**Conclusions:**

Cefepime/taniborbactam was highly active against ENSE with various antimicrobial resistance phenotypes/genotypes. ENSE isolates with cefepime/taniborbactam MIC values ≥ 4 mg/L possessed combinations of β-lactam resistance mechanisms, including β-lactamase genes, as well as alterations in OmpC and/or OmpF and/or PBP3.

## Introduction

Carbapenems such as ertapenem, imipenem, meropenem and doripenem are used both empirically and as directed therapy for infections caused by resistant and MDR pathogens including Enterobacterales.^1–5^ Carbapenems and β-lactam/β-lactamase inhibitors are viewed as effective therapies for infections caused by MDR Enterobacterales.^1,2,6^ However, as carbapenem non-susceptibility increases, clinicians/researchers are seeking carbapenem-sparing regimens such as novel β-lactam/β-lactamase inhibitors (including ceftazidime/avibactam and ceftolozane/tazobactam).^1,2,6,7^ There is an unmet need for novel carbapenem-sparing β-lactam/β-lactamase inhibitors to treat infections caused by MDR Gram-negative bacilli including carbapenem-non-susceptible Enterobacterales.

Cefepime is a parenteral extended-spectrum cephalosporin that has been used clinically for decades.^8^ Taniborbactam (formerly VNRX-5133) is a boronic acid-containing β-lactamase inhibitor that inhibits class A, C and D (serine) β-lactamases, and class B (metallo) β-lactamases, including VIM, NDM, SPM-1 and GIM-1 (but not IMP). Cefepime/taniborbactam is in Phase 3 clinical development for the treatment of complicated urinary tract infection.^8–10^

The current study assessed the *in vitro* activities of cefepime/taniborbactam and comparator antimicrobial agents against ertapenem-non-susceptible Enterobacterales (ENSE) clinical isolates collected from the CANWARD study 2007–19, as well as associations between MIC and various mechanisms of β-lactam resistance identified using WGS.

## Materials and methods

### CANWARD study

CANWARD is a national, ongoing, Public Health Agency of Canada-National Microbiology Laboratory (PHAC-NML)/Canadian Antimicrobial Resistance Alliance (CARA) partnered surveillance study evaluating *in vitro* activities of antimicrobial agents against bacterial pathogens isolated by clinical laboratories from patients attending tertiary care hospitals across Canada.^3^ Each isolate submitted was considered clinically significant by protocols in place at the submitting laboratory. The CANWARD surveillance study sets annual quotas for respiratory, wound, urine and bloodstream isolates and requires isolates be collected consecutively, one per patient, per site of infection, from both in- and outpatients attending emergency rooms, hospital clinics, medical/surgical wards and ICUs each year.^3^ All isolates are shipped to the CANWARD coordinating laboratory (Health Sciences Centre, Winnipeg, Canada) where their identities are confirmed by colonial appearance, spot testing and/or MALDI-TOF MS (Bruker Daltonics, Billerica, MA, USA).^3^ Tertiary care hospitals in 8 of the 10 Canadian provinces participate in the CANWARD surveillance study. The number of tertiary care hospitals participating in the CANWARD surveillance study by year was: 12 in 2007, 10 in 2008, 15 in 2009, 14 in 2010, 15 in 2011, 12 in 2012, 15 in 2013, 13 in 2014, 13 in 2015, 13 in 2016, 14 in 2017, 12 in 2018 and 10 in 2019. The CANWARD surveillance study receives annual approval by the University of Manitoba Research Ethics Board (H2009:059).

### Bacterial isolates

The CANWARD 2007–19 isolate collection contained 18 027 isolates of Enterobacterales of which 179 (0.99%) were ertapenem-non-susceptible (MIC ≥ 1 mg/L).^3^ The 179 ENSE included in the current study were *Enterobacter cloacae* (*n *= 96), *Escherichia coli* (*n *= 26) and 57 other species [*Klebsiella aerogenes* (*n *= 20), *Klebsiella pneumoniae* (*n *= 26), *Klebsiella oxytoca* (*n *= 1), *Serratia marcescens* (*n *= 7), *Citrobacter freundii* (*n *= 2) and *Morganella morganii* (*n *= 1)]. Specimen sources were 43% respiratory, 39% blood, 10% urine and 8% wound.

Fifty-one ertapenem-susceptible (MIC ≤0.5 mg/L) Enterobacterales {*E. cloacae* (*n *= 26), *E. coli* (*n *= 7) and 18 other species [*K. aerogenes* (*n *= 6), *K. pneumoniae* (*n *= 6), *K. oxytoca* (*n *= 1), *S. marcescens* (*n *= 3), *Proteus mirabilis* (*n *= 1) and *C. freundii* (*n *= 1)]} were randomly selected from the CANWARD 2007–19 isolate collection and tested as controls.

### Antimicrobial susceptibility testing

Following two subcultures from frozen stock, the *in vitro* activities of cefepime/taniborbactam (cefepime doubling dilution range 0.03–128 mg/L with taniborbactam fixed at 4 mg/L) and comparator agents were determined by reference CLSI broth microdilution (M07, 11th edition, 2018) using 96-well custom designed microtitre plates.^3,11^ Antimicrobial agents were obtained as laboratory grade powders from their respective manufacturers or commercial sources. Stock solutions were prepared and dilutions made as described by CLSI.^3,11^ MICs were interpreted using CLSI M100 (30th edition, 2020) or FDA breakpoints.^12,13^ Isolates with cefepime/taniborbactam MIC ≤ 8 mg/L were deemed susceptible.

Colony counts were performed to confirm inocula. Quality control was assured using *E. coli* ATCC 25922, *Pseudomonas aeruginosa* ATCC 27853, *E. coli* NCTC 13353 (CTX-M-15) (routine quality control strain used for cefepime/taniborbactam) and *K. pneumoniae* ATCC BAA-1705 (KPC-2, TEM, SHV).

### WGS

ENSE isolates, plus 51 ertapenem-susceptible Enterobacterales controls, were sequenced using the Illumina NextSeq platform. Quality control was assessed using the FastQC tool (https://www.bioinformatics.babraham.ac.uk/projects/fastqc/) and contigs were assembled using SPAdes software.^14^ Sequencing yielded an average of 2 637 395 reads per genome and an average genome coverage of 82×. *De novo* assembly yielded an average contig length and N50 length of 128 583 and 318 077 bp, respectively.

MLST alleles and STs were identified by scanning assembled contigs against available PubMLST databases (https://github.com/tseemann/mlst). Carbapenemases, ESBLs and other β-lactamases were identified using ResFinder 4.0 at an identity threshold of 90%.^15^ Alterations in genes *ompC*/*F* (encoding major porins), *ompK37* (in *K. pneumoniae* only) and *ftsI* (encoding PBP3) were identified by comparing extracted sequences to the appropriate NCBI reference gene. PBP3 alterations of interest included four amino acid insertions after P333, as previously described.^16^ Porin alterations were analysed with Provean v1.1.3 (default settings) to predict those that may have a negative impact on biological protein function.^17^ Only alterations predicted by Provean as having a negative impact on Omp function are discussed further in this study.

### Nucleotide sequence accession numbers

The WGS data reported in this study have been deposited in the NCBI Short Read Archive under BioProject PRJNA736690.

## Results

### Activity of cefepime/taniborbactam against ertapenem-susceptible Enterobacterales and ENSE

The cefepime/taniborbactam MIC_50_ and MIC_90_ were 0.06 mg/L and 0.25 mg/L, respectively (MIC range ≤ 0.03–2 mg/L, an 8-fold potentiation compared with cefepime based on MIC_90_ values) with 100% susceptibility (MIC ≤ 8 mg/L) for the 51 ertapenem-susceptible Enterobacterales used as control isolates (Table 1). Susceptibilities with comparator agents ranged from 84.3% with ceftolozane/tazobactam and piperacillin/tazobactam to 100% with meropenem and meropenem/vaborbactam (Table 1). The cefepime/taniborbactam MIC_50_ and MIC_90_ were 0.5 mg/L and 2 mg/L, respectively (MIC range 0.06–32 mg/L) for the 179 ENSE (Table 1). The presence of taniborbactam at 4 mg/L increased the proportion of ENSE isolates inhibited at ≤ 8 mg/L cefepime to 98.9% compared with 42.5% for cefepime alone (a ≥ 32-fold potentiation compared with cefepime based on MIC_90_ values). For these 179 ENSE, not surprisingly, the susceptibilities to other β-lactam or β-lactam-like comparator agents ranged from lows of 15.1% susceptibility to piperacillin/tazobactam and 20.1% to ceftolozane/tazobactam to highs of 92.2% susceptibility to imipenem/relebactam, 96.1% to meropenem/vaborbactam and 97.8% to ceftazidime/avibactam (Table 1).

**Table 1.** Activity of cefepime/taniborbactam and comparators for various ertapenem-susceptible and various resistance phenotypes of ertapenem non-susceptible Enterobacterales

Phenotype (no. tested)/Antimicrobial agent	MIC (mg/L)			% S	% I	% R
	MIC_50_	MIC_90_	range			
Ertapenem-S (51)						
Cefepime/taniborbactam	0.06	0.25	0.03 to 2	100^a^	—	0
Cefepime	0.12	2	≤ 0.03 to 32	96.1	1.9^b^	2.0
Ceftazidime/avibactam	≤ 0.25	0.5	≤ 0.25 to 4	100	—	0
Ceftolozane/tazobactam	0.5	4	≤ 0.25 to 16	84.3	5.9	9.8
Gentamicin	0.5	2	≤ 0.25 to > 16	94.1	0	5.9
Imipenem	0.5	1	≤ 0.25 to 4	90.2	7.8	2.0
Imipenem/relebactam	0.25	0.5	≤ 0.12 to 4	96.1	1.9	2.0
Levofloxacin	≤ 0.25	1	≤ 0.25 to > 8	86.3	5.9	7.8
Meropenem	≤ 0.06	0.12	≤ 0.06 to 0.5	100	0	0
Meropenem/vaborbactam	≤ 0.06	≤ 0.06	≤ 0.06 to 0.12	100	0	0
Piperacillin/tazobactam	4	32	≤ 0.5 to > 128	84.3	9.8	5.9
Ertapenem-NS (179)						
Cefepime/taniborbactam	0.5	2	0.06 to 32	98.9^a^	—	1.1
Cefepime	4	> 64	0.12 to > 64	42.5	30.1^b^	27.4
Ceftazidime/avibactam	1	4	≤ 0.25 to > 32	97.8	—	2.2
Ceftolozane/tazobactam	16	> 32	≤ 0.25 to > 32	20.1	10.1	69.8
Gentamicin	0.5	> 16	≤ 0.25 to > 16	77.7	2.2	20.1
Imipenem	1	8	≤ 0.25 to > 32	72.1	12.3	15.6
Imipenem/relebactam	0.25	1	≤ 0.12 to 32	92.2	3.3	4.5
Levofloxacin	0.5	> 8	≤ 0.25 to > 8	50.8	7.3	41.9
Meropenem	0.25	8	≤ 0.06 to > 32	81.6	4.4	14.0
Meropenem/vaborbactam	≤ 0.06	0.5	≤ 0.06 to > 32	96.1	1.7	2.2
Piperacillin/tazobactam	128	> 128	1 to > 128	15.1	25.7	59.2
Aztreonam-R (157)						
Cefepime/taniborbactam	0.5	2	0.06 to 32	99.4^a^	—	0.6
Cefepime	4	> 64	0.12 to > 64	36.3	33.8^b^	29.9
Ceftazidime/avibactam	1	4	≤ 0.25 to > 32	98.1	—	1.9
Ceftolozane/tazobactam	16	> 32	0.5 to > 32	11.5	10.2	78.3
Gentamicin	0.5	> 16	≤ 0.25 to > 16	76.4	1.9	21.7
Imipenem	0.5	4	≤ 0.25 to > 32	73.9	11.5	14.6
Imipenem/relebactam	0.25	1	≤ 0.12 to 32	94.9	0.6	4.5
Levofloxacin	0.5	> 8	≤ 0.25 to > 8	50.3	6.4	43.3
Meropenem	0.25	8	≤ 0.06 to > 32	80.9	5.1	14.0
Meropenem/vaborbactam	≤ 0.06	0.5	≤ 0.06 to > 32	96.2	1.9	1.9
Piperacillin/tazobactam	128	> 128	4 to > 128	8.3	25.5	66.2
Cefepime-R (49)						
Cefepime/taniborbactam	1	4	0.12 to 32	95.9^a^	—	4.1
Cefepime	> 64	> 64	16 to > 64	0	0^b^	100
Ceftazidime/avibactam	1	8	≤ 0.25 to > 32	91.8	—	8.2
Ceftolozane/tazobactam	> 32	> 32	1 to > 32	16.3	2.1	75.5
Gentamicin	> 16	> 16	≤ 0.25 to > 16	42.9	2.0	55.1
Imipenem	1	32	≤ 0.25 to > 32	53.1	12.2	34.7
Imipenem/relebactam	0.25	4	≤ 0.12 to 32	85.7	2.1	12.2
Levofloxacin	> 8	> 8	≤ 0.25 to > 8	8.2	4.0	87.8
Meropenem	0.5	32	≤ 0.06 to > 32	55.1	8.2	36.7
Meropenem/vaborbactam	≤ 0.06	8	≤ 0.06 to > 32	87.8	4.0	8.2
Piperacillin/tazobactam	> 128	> 128	4 to > 128	12.2	6.2	81.6
Meropenem-R (25)						
Cefepime/taniborbactam	1	8	0.12 to 32	92.0^a^	—	8.0
Cefepime	32	> 64	0.5 to > 64	8.0	20.0^b^	72.0
Ceftazidime/avibactam	2	> 32	≤ 0.25 to > 32	84.0	—	16.0
Ceftolozane/tazobactam	> 32	> 32	1 to > 32	8.0	0.0	92.0
Gentamicin	1	> 16	≤ 0.25 to > 16	64.0	4.0	32.0
Imipenem	8	> 32	2 to > 32	0.0	12.0	88.0
Imipenem/relebactam	1	16	≤ 0.12 to 32	60.0	8.0	32.0
Levofloxacin	> 8	> 8	0.5 to > 8	16.0	4.0	80.0
Meropenem	16	> 32	4 to > 32	0	0	100
Meropenem/vaborbactam	1	16	≤ 0.06 to > 32	72.0	12.0	16.0
Piperacillin/tazobactam	> 128	> 128	8 to > 128	8.0	8.0	84.0
Piperacillin/tazobactam-R (106)						
Cefepime/taniborbactam	0.5	4	0.06 to 32	98.1^a^	—	1.9
Cefepime	8	> 64	0.25 to > 64	20.8	41.5^b^	37.7
Ceftazidime/avibactam	1	4	≤ 0.25 to > 32	96.2	—	3.8
Ceftolozane/tazobactam	32	> 32	2 to > 32	0.9	1.0	98.1
Gentamicin	0.5	> 16	≤ 0.25 to > 16	70.8	3.7	25.5
Imipenem	1	8	≤ 0.25 to > 32	67.0	13.2	19.8
Imipenem/relebactam	0.25	1	≤ 0.12 to 32	91.5	1.9	6.6
Levofloxacin	1	> 8	≤ 0.25 to > 8	48.1	6.6	45.3
Meropenem	0.25	16	≤ 0.06 to > 32	73.6	6.6	19.8
Meropenem/vaborbactam	≤ 0.06	2	≤ 0.06 to > 32	93.4	2.8	3.8
Piperacillin/tazobactam	> 128	> 128	128 to > 128	0	0	100
Ceftolozane/tazobactam-R (125)						
Cefepime/taniborbactam	0.5	2	0.06 to 32	98.4^a^	—	1.6
Cefepime	4	> 64	0.12 to > 64	28.0	40.0^b^	32.0
Ceftazidime/avibactam	1	4	0.25 to > 32	96.8	—	3.2
Ceftolozane/tazobactam	16	> 32	8 to > 32	0	0	100
Gentamicin	0.5	> 16	≤ 0.25 to > 16	76.0	2.4	21.6
Imipenem	1	8	≤ 0.25 to > 32	71.2	10.4	18.4
Imipenem/relebactam	0.25	1	≤ 0.12 to 32	92.8	1.6	5.6
Levofloxacin	0.5	> 8	≤ 0.25 to > 8	52.0	5.6	42.4
Meropenem	0.25	16	≤ 0.06 to > 32	76.8	4.8	18.4
Meropenem/vaborbactam	≤ 0.06	1	≤ 0.06 to > 32	94.4	2.4	3.2
Piperacillin/tazobactam	> 128	> 128	32 to > 128	0	16.8	83.2
Colistin-R (27)						
Cefepime/taniborbactam	1	4	0.12 to 8	100^a^	—	0
Cefepime	2	> 64	0.25 to > 64	66.7	14.8^b^	18.5
Ceftazidime/avibactam	1	4	0.5 to 4	100	—	0
Ceftolozane/tazobactam	8	> 32	1 to > 32	33.3	7.4	59.3
Gentamicin	1	> 16	≤ 0.25 to > 16	77.8	7.4	14.8
Imipenem	1	8	≤ 0.25 to > 32	51.9	18.5	29.6
Imipenem/relebactam	1	2	≤ 0.12 to 32	70.4	22.2	7.4
Levofloxacin	1	> 8	≤ 0.25 to > 8	40.7	14.9	44.4
Meropenem	0.25	16	0.12 to > 32	81.5	0	18.5
Meropenem/vaborbactam	≤ 0.06	2	≤ 0.06 to 8	96.3	3.7	0
Piperacillin/tazobactam	64	> 128	1 to > 128	22.2	29.7	48.1
Levofloxacin-R (75)						
Cefepime/taniborbactam	1	4	0.06 to 32	97.3^a^	—	2.7
Cefepime	16	> 64	0.25 to > 64	22.7	20.0^b^	57.3
Ceftazidime/avibactam	1	4	≤ 0.25 to > 32	94.7	—	5.3
Ceftolozane/tazobactam	16	> 32	0.5 to > 32	22.7	6.6	70.7
Gentamicin	2	> 16	≤ 0.25 to > 16	56.0	2.7	41.3
Imipenem	1	16	≤ 0.25 to > 32	61.3	10.7	28.0
Imipenem/relebactam	0.25	1	≤ 0.12 to 32	92.0	2.7	5.3
Levofloxacin	> 8	> 8	2 to > 8	0	0	100
Meropenem	0.25	16	≤ 0.06 to > 32	68.0	5.3	26.7
Meropenem/vaborbactam	≤ 0.06	2	≤ 0.06 to > 32	94.7	1.3	4.0
Piperacillin/tazobactam	> 128	> 128	4 to > 128	17.3	18.7	64.0
Imipenem-R (28)						
Cefepime/taniborbactam	1	8	0.12 to 32	92.9^a^	—	7.1
Cefepime	16	> 64	0.25 to > 64	21.4	17.9^b^	60.7
Ceftazidime/avibactam	2	> 32	≤ 0.25 to > 32	89.3	—	10.7
Ceftolozane/tazobactam	> 32	> 32	1 to > 32	17.9	0	82.1
Gentamicin	2	> 16	≤ 0.25 to > 16	64.3	3.6	32.1
Imipenem	8	> 32	4 to > 32	0	0	100
Imipenem/relebactam	1	16	≤ 0.12 to 32	57.1	14.3	28.6
Levofloxacin	8	> 8	≤ 0.25 to > 8	21.4	3.6	75.0
Meropenem	16	> 32	0.5 to > 32	10.7	10.7	78.6
Meropenem/vaborbactam	0.5	16	≤ 0.06 to > 32	75.0	10.7	14.3
Piperacillin/tazobactam	> 128	> 128	1 to > 128	17.9	7.1	75.0
Trimethoprim/sulfamethoxazole-R (60)						
Cefepime/taniborbactam	0.5	4	0.06 to 32	96.7^a^	—	3.3
Cefepime	32	> 64	0.25 to > 64	21.7	15.0^b^	63.3
Ceftazidime/avibactam	1	8	≤ 0.25 to > 32	93.3	—	6.7
Ceftolozane/tazobactam	32	32	0.5 to > 32	25.0	6.7	68.3
Gentamicin	16	> 16	≤ 0.25 to > 16	41.7	6.6	51.7
Imipenem	1	16	≤ 0.25 to > 32	53.3	18.4	28.3
Imipenem/relebactam	0.25	2	≤ 0.12 to 32	88.3	5.0	6.7
Levofloxacin	> 8	> 8	≤ 0.25 to > 8	6.7	10.0	83.3
Meropenem	0.5	32	≤ 0.06 to > 32	61.7	8.3	30.0
Meropenem/vaborbactam	≤ 0.06	2	≤ 0.06 to > 32	95.0	0	5.0
Piperacillin/tazobactam	> 128	> 128	4 to > 128	20.0	13.3	66.7
Amoxicillin/clavulanate-R (171)						
Cefepime/taniborbactam	0.5	2	0.06 to 32	98.8^a^	—	1.2
Cefepime	4	> 64	0.12 to > 64	42.7	31.0^b^	26.3
Ceftazidime/avibactam	1	4	≤ 0.25 to > 32	97.7	—	2.3
Ceftolozane/tazobactam	16	> 32	0.5 to > 32	16.4	10.5	73.1
Gentamicin	0.5	> 16	≤ 0.25 to > 16	77.8	2.3	19.9
Imipenem	1	8	≤ 0.25 to > 32	70.8	12.8	16.4
Imipenem/relebactam	0.25	1	≤ 0.12 to 32	91.8	3.5	4.7
Levofloxacin	0.5	> 8	≤ 0.25 to > 8	52.0	7.1	40.9
Meropenem	0.25	8	≤ 0.06 to > 32	80.7	4.7	14.6
Meropenem/vaborbactam	≤ 0.06	0.5	≤ 0.06 to > 32	95.9	1.8	2.3
Piperacillin/tazobactam	128	> 128	1 to > 128	11.7	26.3	62.0

S, susceptible; I, intermediate; R, resistant; NS, non-susceptible.

a Proportion of isolates inhibited at ≤ 8 mg/L.

b Interpreted using SDD breakpoint (4–8 mg/L; CLSI M100 31st edition).

### Activity of cefepime/taniborbactam against additional resistant phenotypes

The ENSE isolates were extensively cross-resistant to other antimicrobial agents. For example, 95.5% (171/179), 87.7% (157/179) and 69.8% (125/179) of isolates were also resistant to amoxicillin/clavulanate (*n *= 171), aztreonam (*n *= 157) and ceftolozane/tazobactam (*n *= 125), respectively, attesting to the challenging nature of this collection of isolates. Cefepime/taniborbactam was highly active against ENSE isolates with various antimicrobial resistance phenotypes including: amoxicillin/clavulanate-resistant (MIC_50_ 0.5 mg/L; MIC_90_ 2 mg/L), aztreonam-resistant (MIC_50_ 0.5 mg/L; MIC_90_ 2 mg/L), cefepime-resistant (MIC_50_ 1 mg/L; MIC_90_ 4 mg/L), ceftolozane/tazobactam-resistant (MIC_50_ 0.5 mg/L; MIC_90_ 4 mg/L), colistin-resistant (MIC_50_ 1 mg/L; MIC_90_ 4 mg/L), imipenem-resistant (MIC_50_ 1 mg/L; MIC_90_ 8 mg/L), levofloxacin-resistant (MIC_50_ 1 mg/L; MIC_90_ 4 mg/L), meropenem-resistant (MIC_50_ 1 mg/L; MIC_90_ 8 mg/L), piperacillin/tazobactam-resistant (MIC_50_ 0.5 mg/L; MIC_90_ 4 mg/L) and trimethoprim/sulfamethoxazole-resistant (MIC_50_ 0.5 mg/L; MIC_90_ 4 mg/L) with susceptibilities ranging from 92%–100% (Table 1).

Table 2 demonstrates the MICs of cefepime/taniborbactam and select comparators for a limited number of imipenem/relebactam-resistant, ceftazidime/avibactam-resistant and meropenem/vaborbactam-resistant isolates. Cefepime/taniborbactam demonstrated MICs of 0.5–4 mg/L for 6 of 8, 2 of 4 and 2 of 4 isolates resistant to imipenem/relebactam, ceftazidime/avibactam and meropenem/vaborbactam, respectively (Table 2). Cefepime/taniborbactam was the most active agent tested against imipenem/relebactam and meropenem/vaborbactam-resistant isolates (Table 2).

**Table 2.** Cefepime/taniborbactam and comparator MIC distributions for various resistant phenotypes of Enterobacterales

Phenotype (*n*)	MIC (mg/L)									
	≤ 0.25	0.5	1	2	4	8	16	32	64	> 64
Imipenem/relebactam-R (8)										
Cefepime		1				1	2			4
Cefepime/taniborbactam		1	1	2	2			2		
Ceftazidime/avibactam		1		1	1	2		3^b^		
Imipenem/relebactam					4	1	1	2		
Meropenem/vaborbactam	1^a^					3	2	2^b^		
Ceftazidime/avibactam-R (4)										
Cefepime									1	3
Cefepime/taniborbactam			2					2		
Ceftazidime/avibactam								4^b^		
Imipenem/relebactam		1			1		1	1		
Meropenem/vaborbactam					1		1	2^b^		
Meropenem/vaborbactam-R (4)										
Cefepime							1			3
Cefepime/taniborbactam			1	1				2		
Ceftazidime/avibactam					1			3^b^		
Imipenem/relebactam					1	1	1	1		
Meropenem/vaborbactam							2	2^b^		

a Shown are numbers for the lowest common concentration; actual MICs of some isolates may be lower than indicated.

b Shown are numbers for the highest common concentration; actual MICs of some isolates may be higher than indicated.

### β-Lactam resistance mechanisms

Of the 179 ENSE, 8.9% (*n *= 16) possessed a carbapenemase, 21.8% (*n *= 39) possessed an ESBL and 97.2% (*n *= 174) possessed other β-lactamase gene(s) (Table S1, available as Supplementary data at *JAC-AMR* Online). Six isolates possessed a carbapenemase, an ESBL and other β-lactamase genes. All isolates with carbapenemases and 97.4% of isolates with an ESBL (*n *= 38) also possessed truncated or altered Omp proteins. Overall, 88.3% (*n *= 158) of ENSE had at least one altered/truncated porin. Only two isolates (1.1%) possessed previously described alterations in PBP3, one each of Y333_R334insYRIK and Y333_R334insYRIN.

Of the 51 ertapenem-susceptible control isolates, 84.3% (*n *= 43) had at least one altered/truncated porin gene and 74.5% (*n *= 38) possessed a non-carbapenemase, non-ESBL β-lactamase gene (Table S1). However, no carbapenemase genes or PBP3 alterations were found within these isolates. Three isolates (5.9%) possessed an ESBL gene, though these were only within isolates at the highest level of ertapenem susceptibility (MIC = 0.5 mg/L).

### Specific gene content of ENSE with cefepime/taniborbactam MICs ≥ 4 mg/L

Although the CLSI susceptible, dose-dependent (SDD) breakpoint for cefepime/taniborbactam for Enterobacterales is ≤ 8 mg/L, this analysis also examined the characteristics of isolates within the cefepime SDD range (cefepime/taniborbactam MICs of 4–8 mg/L) as well as above 8 mg/L. Only 7.2% (*n *= 13) of ENSE isolates demonstrated a cefepime/taniborbactam MIC ≥ 4 mg/L, including seven *K. pneumoniae*, three *E. coli,* two *E. cloacae* and one *S. marcescens* (Table 3). Each of the 13 isolates possessed at least one β-lactamase gene and at least one altered or truncated Omp (including OmpK37 for one *K. pneumoniae* isolate) (Table 3). Only two isolates with cefepime/taniborbactam MIC ≥ 4 mg/L possessed a carbapenemase (OXA-48; NDM-5 + OXA-181); more commonly, isolates possessed an ESBL with multiple additional β-lactamase genes. Seven isolates possessed a truncated Omp, while eight had Omps with insertions of two or more amino acids (with or without accompanying deletions) (Table 3). Only one isolate possessed a four amino acid insertion after P333 in PBP3 (see below). Frequently these 13 isolates also demonstrated elevated MICs or resistance to ceftazidime/avibactam, imipenem/relebactam and meropenem/vaborbactam (Table 3).

**Table 3.** Expanded gene content of 13 ENSE isolates with cefepime/taniborbactam MICs ≥ 4 mg/L

FTB MIC/Organism (ST)	MIC (mg/L)		CPs	ESBLs	AmpC/Class C βL	Other βLs^a^	Porin alterations^b^		PBP3 insertion
	CPM	CZA/IMR/MEV					OmpC (OmpK36)	OmpF (OmpK35)	
4 mg/L									
* E. cloacae* (ST141)	8	8/4/8	—	—	ACT-7	—	G104A, G358Q, L359F	—	—
* E. cloacae* (ST50)	32	8/0.5/1	—	SHV-12	ACT-15	—	g.93_97dupATTAG → frameshift, premature stop codon (truncated 33aa protein)	g.398delC → frameshift, premature stop codon (truncated 167aa protein)	—
* E. coli* (ST68)	16	2/0.5/≤ 0.06	—	—	CMY-10	OXA–4, OXA–47	N165D, F182_R195delinsMTTNGRDDVFE, D208N, D225W	g.470_473delGCGT → frameshift, premature stop codon (truncated 171aa protein)	—
KPN (ST147)	> 64	1/≤ 0.12/≤ 0.06	—	CTX-M-15	DHA-1	OXA-1, OXA–10, SHV-11, TEM-1B	A183_T184insLSP, T222L	—	—
KPN (ST391)	> 64	2/1/0.12	—	CTX-M-15	—	OXA-1, SHV-11, TEM-1B	A190W, N304delinsER	—	—
KPN (ST147)	> 64	0.5/0.25/0.5	—	CTX-M-15	—	OXA-1, SHV-11, TEM-1B	g.78_79insT → frameshift, premature stop codon (truncated 26aa protein)	—	—
KPN (ST14)	> 64	2/0.25/0.5	—	CTX-M-15	—	OXA-1, OXA-9, SHV-28, TEM-1A	G134_D135insDG, A190W, N304delinsER	g.676_694delAAAGCCGAAGCCTGGGGCGA → frameshift, premature stop codon (truncated 246aa protein)	—
KPN (ST101)	> 64	2/4/8	OXA-48	CTX-M-15	—	OXA-1, SCO-1, SHV-1, TEM-1A	G134_D135insDG, A190W, N304delinsER	g.185delG → frameshift, premature stop codon (truncated 62aa protein)	—
SER (NA)	8	4/2/2	—	—	SST-1	—	—	I360T	—
8 mg/L									
KPN (ST14)	> 64	4/0.5/0.5	—	CTX-M-15	—	OXA-1, OXA-9, SHV-28, TEM-1A	G134_D135insDG, A190W, N304delinsER	g.676_694delAAAGCCGAAGCCTGGGGCGA → frameshift, premature stop codon (truncated 246aa protein)	—
KPN (ST45)	> 64	2/2/4	—	CTX-M-15	—	OXA-1, SHV-1, TEM-1B	—	—	—
32 mg/L									
* E. coli* (ST405)	> 64	> 32/4/16	—	CTX-M-71	—	—	N165D, F182_R195delinsMTTNGRDDVFE, D208N, D225W	g.753_756dupGAAC → frameshift, premature stop codon (truncated 256aa protein)	—
* E. coli* (ST361)	> 64	> 32/32/> 32	NDM-5, OXA-181	—	—	TEM-1B	D192G	—	P333_Y334insYRIN

FTB, cefepime/taniborbactam; CPM, cefepime; CZA, ceftazidime/avibactam; IMR, imipenem/relebactam; MEV, meropenem/vaborbactam; KPN, *K. pneumoniae*; SER, *S. marcescens*; CP, carbapenemase; βL, β-lactamase.

a Includes all other β-lactamases that are not carbapenemases, ESBLs or class C enzymes.

b Including truncations due to a premature stop codon, or alterations predicted by Provean to have a negative impact on biological protein function.

c KPN isolate contained N230G and M233_R239delinsQHYTHTERYAK in OmpK37.

Of note, two *E. coli* isolates had a cefepime/taniborbactam MIC of 32 mg/L (cefepime MICs > 64 mg/L). One possessed NDM-5, OXA-181 and TEM-1B, an OmpC alteration (D192G) and P333_Y334insYRIN in PBP3. The second contained CTX-M-71, a truncated OmpF due to a four amino acid duplication and a large alteration in OmpC (F182_R195delinsMTTNGRDDVFE) (Table 3). These isolates were from different clonal groups (ST361 and ST405). These two isolates were also concomitantly resistant to ceftazidime/avibactam, imipenem/relebactam and meropenem/vaborbactam (Table 3).

### Cefepime/taniborbactam and cefepime MIC distributions for ENSE with known carbapenemase, ESBL and AmpC/Class C β-lactamase genes

In 16 carbapenemase-producing strains, cefepime/taniborbactam MICs were significantly lower than cefepime MICs (Table 4). Cefepime/taniborbactam MICs for isolates containing KPC-2/KPC-3, NDM-1 and OXA-48/OXA-181/OXA-232 were 0.12–4 mg/L in comparison to 2 to > 64 mg/L for cefepime (Table 4). One NDM-1-containing strain was resistant to imipenem/relebactam, ceftazidime/avibactam and meropenem/vaborbactam, but was provisionally susceptible to cefepime/taniborbactam (MIC 1 mg/L). Cefepime/taniborbactam MICs were also significantly lower than cefepime MICs in 39 ESBL-producing strains, where MICs for 92.3% (36/39) isolates containing CTX-M- and SHV-type ESBLs were 0.06–4 mg/L in comparison to 1 to > 64 mg/L for cefepime (Table 5). Cefepime/taniborbactam demonstrated similar activity to ceftazidime/avibactam against the 39 ESBL-producing strains (Table 5). Cefepime/taniborbactam MICs were also significantly lower than cefepime MICs for 139 AmpC/Class C (no carbapenemase gene) producing strains, with cefepime/taniborbactam activity greater than ceftazidime/avibactam and similar to imipenem/relebactam (Table 6). Cefepime/taniborbactam demonstrated greater activity than cefepime against 22 ENSE isolates with OXA-family genes (but no carbapenemase genes) (Table 7).

**Table 4.** Cefepime/taniborbactam and comparator MIC distributions for 16 ENSE isolates with carbapenemase genes

Carbapenemase (organism)	0.12	0.25	0.5	1	2	4	8	16	32	64	> 64	Total
KPC-2 (all *K. pneumoniae*)												4
Cefepime					1		1		2			
Cefepime/taniborbactam	1	1	2									
KPC-3^a^												6
Cefepime								2	1		3	
Cefepime/taniborbactam	2	3		1								
NDM-1 + OXA-232 (all *K. pneumoniae*)										2		
Cefepime										1	1	
Cefepime/taniborbactam				2								
NDM-5 + OXA-181 (*E. coli*)^b^												1
Cefepime											1	
Cefepime/taniborbactam									1			
OXA-48 (*K. pneumoniae*)												1
Cefepime											1	
Cefepime/taniborbactam						1						
OXA-181 (*K. pneumoniae*)												1
Cefepime									1			
Cefepime/taniborbactam	1											
SME-3 (*S. marcescens*)												1
Cefepime			1									
Cefepime/taniborbactam			1									
All isolates with carbapenemase genes												16
Cefepime			1		1		1	2	4	1	6	
Cefepime/taniborbactam	4	4	3	3		1			1			
Ceftazidime/avibactam		3^c^	3	3	4				3^d^			
Imipenem/relebactam	3^c^	2	6	1		1		1	2			
Meropenem/vaborbactam	11^c^	1				1	1		2^d^			

a Includes two E. coli, two K. pneumoniae, one K. oxytoca and one S. marcescens.

b This isolate also possessed P333_Y334insYRIN in PBP3.

c Shown are numbers for the lowest common concentration; actual MICs of some isolates may be lower than indicated.

d Shown are numbers for the highest common concentration; actual MICs of some isolates may be higher than indicated.

**Table 5.** Cefepime/taniborbactam and comparator MIC distributions for 33 ENSE isolates with ESBL genes but no carbapenemase genes

ESBL (organism)	≤ 0.03	0.06	0.12	0.25	0.5	1	2	4	8	16	32	64	> 64	Total
CTX-M-3 + SHV-27 (*K. pneumoniae*)														1
Cefepime													1	
Cefepime/taniborbactam						1								
CTX-M-14 (*E. coli*)														1
Cefepime													1	
Cefepime/taniborbactam			1											
CTX-M-15^a^														22
Cefepime										1			21	
Cefepime/taniborbactam				2	5	6	3	4	2					
CTX-M-67 (*E. coli*)														1
Cefepime									1					
Cefepime/taniborbactam		1												
CTX-M-71 (*E. coli*)^b^														1
Cefepime													1	
Cefepime/taniborbactam											1			
SHV-12 (*E. cloacae*)														7
Cefepime						1		2		2	2			
Cefepime/taniborbactam			1	3		2		1						
All isolates with ESBL genes (no carbapenemase genes)														33
Cefepime						1		2	1	3	2		24	
Cefepime/taniborbactam		1	2	5	5	9	3	5	2		1			
Ceftazidime/avibactam				2^c^	11	10	7	1	1		1^d^			
Imipenem/relebactam			10^c^	13	5	3	1	1						
Meropenem/vaborbactam		18^c^	5	2	3	2	1	1		1				

a Includes 11 K. pneumoniae, 10 E. coli and 1 E. cloacae.

b This isolate also possesses F182_R195delinsMTTNGRDDVFE in OmpC and a truncated OmpF.

c Shown are numbers for the lowest common concentration; actual MICs of some isolates may be lower than indicated.

d Shown are numbers for the highest common concentration; actual MICs of some isolates may be higher than indicated.

**Table 6.** Cefepime/taniborbactam and comparator MIC distributions for 139 ENSE isolates with AmpC/Class C β-lactamase genes but no carbapenemase genes

Class C β-lactamase family (organism)	≤ 0.03	0.06	0.12	0.25	0.5	1	2	4	8	16	32	64	> 64	Total
*ampC* (*K. aerogenes*)														20
Cefepime			1	4	6	4	3			2				
Cefepime/taniborbactam		3	8	2	2	2	3							
ACT family (*E. cloacae*)														84
Cefepime				3	6	10	13	28	17	2	2		3	
Cefepime/taniborbactam			5	33	21	16	7	2						
ACT family + DHA family (*E. cloacae*)														2
Cefepime					1	1								
Cefepime/taniborbactam				1		1								
ACT family + FOX family (*E. cloacae*)														1
Cefepime								1						
Cefepime/taniborbactam			1											
CMH family (*E. cloacae*)														1
Cefepime							1							
Cefepime/taniborbactam						1								
CMY family^a^														9
Cefepime							2	3	2	1	1			
Cefepime/taniborbactam				5	1	1	1	1						
DHA family^b^														8
Cefepime				3									5	
Cefepime/taniborbactam		1	1	1	1	3		1						
EC family (*E. coli*)														1
Cefepime				1										
Cefepime/taniborbactam				1										
MIR family (*E. cloacae*)														8
Cefepime			1	2	1	4								
Cefepime/taniborbactam		3	2	2		1								
SRT/SST family (*S. marcescens*)														5
Cefepime					1	1	2		1					
Cefepime/taniborbactam					2	2		1						
All isolates with Class C β-lactamase genes (no carbapenemase genes)														139
Cefepime			2	13	15	20	21	32	20	5	3		8	
Cefepime/taniborbactam		7	17	45	27	27	11	5						
Ceftazidime/avibactam				9^c^	34	61	22	8	5					
Imipenem/relebactam			43^c^	63	15	10	5	2	1					
Meropenem/vaborbactam		106^c^	20	2	5	1	2		2	1				

a Includes seven *E. coli* and two *C. freundii*.

b Includes six K. pneumoniae, one E. coli and one M. morganii.

c Shown are numbers for the lowest common concentration; actual MICs of some isolates may be lower than indicated.

**Table 7.** Cefepime/taniborbactam and comparator MIC distributions for 22 ENSE with OXA-family genes but no carbapenemase genes

OXA gene (organism)	≤ 0.03	0.06	0.12	0.25	0.5	1	2	4	8	16	32	64	> 64	Total
OXA-1^a^														17
Cefepime					1		1			1			14	
Cefepime/taniborbactam				1	4	6	3	2	1					
OXA-1 + OXA-9 (*K. pneumoniae*)														2
Cefepime													2	
Cefepime/taniborbactam								1	1					
OXA-1 + OXA-10 (*K. pneumoniae*)														1
Cefepime													1	
Cefepime/taniborbactam								1						
OXA-4 + OXA-47 (*E. coli*)														1
Cefepime										1				
Cefepime/taniborbactam								1						
OXA-10 (*E. cloacae*)														1
Cefepime							1							
Cefepime/taniborbactam					1									
All isolates with OXA genes (no carbapenemase genes)														22
Cefepime					1		2			2			17	
Cefepime/taniborbactam				1	5	6	3	5	8					
Ceftazidime/avibactam				1^b^	8	4	8	1						
Imipenem/relebactam			8^b^	8	3	2	1							
Meropenem/vaborbactam		14^b^	3	1	3			1						

a Includes eight *K. pneumoniae*, six *E. coli* and three *E. cloacae*.

b Shown are numbers for the lowest common concentration; actual MICs of some isolates may be lower than indicated here.

## Discussion

Of the 18 027 Enterobacterales isolates collected from CANWARD from 2007 to 2019, we obtained and tested the 0.99% (179/18 027) of isolates that were ENSE (ertapenem MIC ≥1 mg/L).^3^ In this study we assessed the activity of cefepime/taniborbactam against this highly selected cohort of ENSE clinical isolates which using WGS were found to contain carbapenemase genes (8.9%), ESBL genes (21.8%) and other β-lactamases (e.g. AmpC) (97.2%) as well as porin alterations (88.3%) and insertions in PBP3 (1.1%) (Table 3, Table S1). The low number of ENSE isolates with a carbapenemase (8.9%) may reflect that we studied ertapenem-non-susceptible isolates rather than meropenem-resistant isolates. Not surprisingly, the 179 ENSE isolates demonstrated low susceptibilities to other β-lactam and β-lactam-like agents (Table 1). Against this MDR cohort, cefepime/taniborbactam demonstrated a > 64-fold reduction in MIC_90_ compared with cefepime (MIC_90_, 2 mg/L versus > 64 mg/L, respectively) and was active against subsets of isolates with various β-lactam and non-β-lactam antimicrobial resistance phenotypes (Table 1). These data are consistent with Hamrick *et al.*^10^ who reported that the addition of taniborbactam (fixed concentration at 4 mg/L) potentiated cefepime activity 8- to > 1024-fold.

As previously stated, taniborbactam—a boronic acid-containing β-lactamase inhibitor—inhibits class A, C and D (serine) β-lactamases and class B (metallo) β-lactamases, including VIM, NDM, SPM-1 and GIM-1 (but not IMP).^8–10^ Our data show that cefepime/taniborbactam (MIC_50_ 1 mg/L; MIC_90_ 4 mg/L), is significantly more active than cefepime (MIC_50_ and MIC_90_ ≥ 64 mg/L) against ESBL-producing Enterobacterales (92.3% of isolates containing CTX-M- and SHV-type ESBLs) with MICs of 0.06–4 mg/L. These data are consistent with previous reports including Hamrick *et al.*^10^ who reported cefepime/taniborbactam MIC_50_/MIC_90_ of 0.06/0.5 mg/L for Enterobacterales ESBL/AmpC producers (cefepime MIC_50_/MIC_90_ of 8/128 mg/L). Wang *et al.*^18^ reported cefepime/taniborbactam MIC_50_/MIC_90_s of 0.03/0.12, 0.06/ 0.25 and 0.12/1 mg/L for ESBL-, plasmid-mediated AmpC- and ESBL with AmpC-producing Enterobacterales isolates, respectively, with corresponding cefepime MIC_50_/MIC_90_ of 8/32, 0.12/2 and 32/256 mg/L. Kloezen *et al.*^19^ reported a cefepime/taniborbactam MIC_90_ ≤ 0.5 mg/L for ESBL producers with median cefepime MIC reductions of 5–9 doubling dilutions in the presence of taniborbactam, depending on the Enterobacterales ESBL genotype.

Our data show that cefepime/taniborbactam is significantly more active than cefepime against carbapenemase-producing Enterobacterales, demonstrating MICs of 0.12–4 mg/L for 93.8% isolates containing KPC, NDM, OXA-48 (OXA-48 like) carbapenemase genes. Our data are consistent with prior data including those of Hamrick *et al.*^10^ who reported cefepime/taniborbactam MIC_50_/MIC_90_ of 0.5/2 mg/L for carbapenemase-producing Enterobacterales containing KPC, NDM, VIM and OXA-48/48-like genes (corresponding cefepime MIC_50_/_90_ 64/> 256 mg/L). Wang *et al.*^18^ reported cefepime/taniborbactam MIC_50_/MIC_90_s of 2/8, 16/64 and 4/32 mg/L against KPC- and NDM-producing Enterobacterales and non-carbapenemase-producing CRE with corresponding cefepime MIC_50_/MIC_90_ for all organisms of > 256/> 256 mg/L. Consistent with our data, Mushtaq *et al.*^9^ reported that cefepime/taniborbactam was active against carbapenemase-producing Enterobacterales containing KPC, NDM, VIM, OXA-48 (OXA-48 like) but not IMP genes. Kloezen *et al.*^19^ reported a cefepime/taniborbactam of MIC_90_ ≤ 1 mg/L for KPC and VIM producers with median cefepime MIC reductions of 7–10 doubling dilutions depending on the Enterobacterales carbapenemase genotype. Piccirilli *et al.*^20^ reported cefepime/taniborbactam MIC_50_/MIC_90_ of 1/4 mg/L against Enterobacterales harbouring a variety of NDM and VIM carbapenemases.^17^ However, cefepime/taniborbactam was not active against strains harbouring IMP, demonstrating MICs of 128 mg/L.^20^

Abdelraouf *et al.*^21^ demonstrated that the *in vitro* activity observed with cefepime/taniborbactam against resistant Enterobacterales was translated to the *in vivo* setting. Using a cefepime/taniborbactam human-simulated regimen equivalent to 2 g/0.5 g q8 h administered as a 2 h infusion (which is the dose used in clinical trials) in mice, against 26 clinical Enterobacterales expressing ESBLs, plasmid-mediated AmpC and/or class A (KPC) or D carbapenemases (OXA-48), the combination exerted potent *in vivo* activity (> 1 log_10_ killing among all the isolates examined with cefepime/taniborbactam MICs up to 16 mg/L) against cefepime-resistant isolates, including serine-carbapenemase producers.

Although the number of isolates tested in the present study was small, we report that cefepime/taniborbactam demonstrated MICs of 0.5–4 mg/L for 75% (6 of 8), 50% (2 of 4) and 50% (2 of 4) of isolates resistant to imipenem/relebactam, ceftazidime/avibactam and meropenem/vaborbactam, respectively. In our study, one isolate containing NDM-1 was resistant to imipenem/relebactam, ceftazidime/avibactam and meropenem/vaborbactam, but was provisionally susceptible to cefepime/taniborbactam (MIC 1 mg/L). This is not surprising as, unlike ceftazidime/avibactam and meropenem/vaborbactam, cefepime/taniborbactam inhibits Enterobacterales with MBL NDM and VIM as well as serine-β-lactamases KPC and OXA-48.^7,9,10,20,22^

Only 13 (7.2%) ENSE isolates demonstrated a cefepime/taniborbactam MIC ≥ 4 mg/L. The 13 isolates represented both microbiological species diversity (7 *K. pneumoniae*, 3 *E. coli*, 2 *E. cloacae* and 1 *S. marcescens*) and clonal diversity (STs). All isolates possessed combinations of β-lactam resistance mechanisms, including a carbapenemase and/or ESBL and/or other β-lactamase genes, as well as alterations in OmpC and/or OmpF (reduced uptake into the periplasmic space) and/or PBP3 (reduced binding to target site) (Table 3). Wang *et al.*^18^ analysed 29 NDM-5-producing *E. coli* isolates from China with cefepime/taniborbactam MICs > 8 mg/L (taniborbactam fixed at 4 mg/L) and documented the presence of PBP3 mutations in 28/29 isolates. A variety of different mutations in PBP3 were documented.^15^ Unfortunately, other (non-PBP3 and non-β-lactamase-mediated) resistance mechanisms, such as porin changes or efflux pump expression, were not characterized in that study. Mushtaq *et al.*^9^ analysed Enterobacterales with cefepime/taniborbactam MICs > 8 mg/L (taniborbactam fixed at 4 mg/L) (*E. coli n *= 15, *Klebsiella* spp. *n *= 19 and *Enterobacter* spp. *n *= 1). These researchers noted both genetic diversity (a variety of STs) as well as no universal resistance mechanism in all isolates but rather combinations of carbapenemases (e.g. NDM-5, NDM-7) along with PBP3 insertions (e.g. after amino acid 333), and/or porin changes (e.g. OmpF).^9^ Kloezen *et al.*^19^ analysed three isolates of Enterobacterales with cefepime/taniborbactam MICs > 4 mg/L (taniborbactam fixed at 4 mg/L). One isolate harboured a VIM gene while the other two carried VIM-1, CMY-13 and *qnrA1* genes. The authors concluded that the presence of VIM and AmpC may reduce cefepime/taniborbactam activity against Enterobacterales. The presence of other underlying resistance mechanisms such as porin alterations, which may reduce periplasmic uptake, or target site binding to PBP3 or efflux were not assessed.

There are limitations to the data presented here that deserve attention. Only a limited number of isolates resistant to ceftazidime/avibactam, imipenem/relebactam and meropenem/vaborbactam were available for testing, thus the promising results showing cefepime/taniborbactam activity against ceftazidime/avibactam-, imipenem/relebactam- and meropenem/vaborbactam-resistant Enterobacterales need to be confirmed by others. In addition, it should be mentioned that the results of our WGS provide genetic associations with phenotypic resistance but have not been proven to result in MIC increases or resistance using complementation studies. Finally, though we assessed β-lactam resistance, porin alterations (which may or may not affect periplasmic uptake) and putative binding to the target site (PBP3), we did not assess efflux pump expression, which is known to confer increased cefepime MICs in Enterobacterales and may affect cefepime/taniborbactam activity.^8^

In summary, the current study demonstrated that cefepime/taniborbactam was highly active against whole genome sequenced ENSE isolates with various antimicrobial resistance phenotypes/genotypes. ENSE isolates with cefepime/taniborbactam MIC values ≥ 4 mg/L possessed combinations of β-lactam resistance mechanisms, including a carbapenemase and/or ESBL and/or other β-lactamase genes, as well as alterations in OmpC and/or OmpF and/or PBP3.

## Supplementary Material

dlab197_Supplementary_Data

## Supplementary data

Table S1 is available as Supplementary data at *JAC-AMR* Online.
