# Supplementary material for: Activity of cefepime/taniborbactam and comparators against whole genome sequenced ertapenem-non-susceptible Enterobacterales clinical isolates: CANWARD 2007–19
Source: JAC Antimicrob Resist. 2022 Feb 7;4(1):dlab197. doi: 10.1093/jacamr/dlab197 (PMC8826793; doi:10.1093/jacamr/dlab197)
Supplement: dlab197_Supplementary_Data [file dlab197_supplementary_data.docx]

**Supplementary data**

**Table S1.** Study isolates and associated metadata, abbreviated MIC data for the agents tested and molecular characterization data

| **Stock** | **Organism ^a^** | **Source ^b^** | **MIC (mg/L)** | | | | | | | | | | | | **β-Lactamase Genes** | | | |
| --- | --- | --- | --- | --- | --- | --- | --- | --- | --- | --- | --- | --- | --- | --- | --- | --- | --- | --- |
|  |  |  | **FTB** | **CPM** | **CZA** | **C/T** | **ETP** | **GEN** | **IMI** | **IMR** | **LEV** | **MER** | **MEV** | **PTZ** | **CP** | **ESBL** | **AmpC/Class C** | **All Other ^c^** |
| 75560 | EC | U | 0.06 | 0.06 | </= 0.25 | </= 0.25 | 0.5 | 0.5 | </= 0.25 | 0.25 | </= 0.25 | </= 0.06 | </= 0.06 | 2 | - | - | blaEC-5 | - |
| 76837 | KP | B | 0.03 | </= 0.03 | </= 0.25 | </= 0.25 | </= 0.25 | </= 0.25 | </= 0.25 | </= 0.12 | </= 0.25 | </= 0.06 | </= 0.06 | 2 | - | - | - | blaSHV-1 |
| 78967 | EC | W | 0.25 | 8 | 2 | >32 | 2 | 0.5 | 2 | 0.25 | 1 | 0.12 | </= 0.06 | >128 | - | - | blaCMY-2 | blaTEM-1C |
| 80101 | EC | U | 0.06 | 0.06 | </= 0.25 | 0.5 | </= 0.25 | 1 | </= 0.25 | 0.25 | </= 0.25 | </= 0.06 | </= 0.06 | 2 | - | - | - | - |
| 80116 | Eclo | W | 2 | 8 | 2 | 32 | 16 | 0.5 | 4 | 0.25 | 0.5 | 2 | 0.5 | >128 | - | - | blaACT-7 | - |
| 80513 | KA | R | 0.06 | 0.06 | </= 0.25 | 0.5 | </= 0.25 | </= 0.25 | 2 | 1 | </= 0.25 | </= 0.06 | </= 0.06 | 4 | - | - | - | - |
| 80828 | Eclo | U | 0.12 | 0.25 | 0.5 | 1 | </= 0.25 | </= 0.25 | </= 0.25 | </= 0.12 | </= 0.25 | </= 0.06 | </= 0.06 | 4 | - | - | blaACT-14 | - |
| 80960 | EC | B | 0.25 | >64 | </= 0.25 | 2 | 1 | 1 | </= 0.25 | </= 0.12 | >8 | 0.12 | </= 0.06 | 4 | - | blaCTX-M-15 | - | blaTEM-1B |
| 81148 | Eclo | U | 0.12 | 2 | 1 | 8 | 1 | </= 0.25 | 0.5 | 0.5 | </= 0.25 | 0.25 | </= 0.06 | 64 | - | - | blaACT-16 | - |
| 82780 | Eclo | B | 0.03 | 0.25 | 0.5 | 0.5 | </= 0.25 | 0.5 | 1 | 0.25 | </= 0.25 | </= 0.06 | </= 0.06 | 2 | - | - | blaACT-3 | - |
| 82940 | EC | B | 4 | 16 | 2 | 8 | 2 | 2 | 0.5 | 0.5 | 8 | 0.5 | </= 0.06 | >128 | - | - | blaCMY-10 | blaOXA-4, blaOXA-47 |
| 83128 | Eclo | B | 1 | 4 | 4 | 32 | 2 | 0.5 | 0.5 | 0.5 | 1 | 0.25 | </= 0.06 | >128 | - | - | blaACT-15 | - |
| 83384 | Eclo | B | 1 | 4 | 1 | 32 | 1 | 0.5 | </= 0.25 | </= 0.12 | 0.5 | 0.12 | </= 0.06 | 128 | - | - | blaACT-15 | - |
| 83848 | KP | B | 1 | 1 | 0.5 | 1 | 1 | </= 0.25 | 1 | 1 | 0.5 | 0.25 | </= 0.06 | 8 | - | - | - | blaSHV-1 |
| 84168 | Eclo | B | 1 | 8 | 2 | 32 | 1 | 1 | 0.5 | 0.25 | 4 | 0.12 | </= 0.06 | >128 | - | - | blaACT-7 | blaTEM-1B |
| 84178 | Eclo | B | 2 | 2 | 0.5 | 2 | 4 | 0.5 | 2 | 0.25 | 8 | 0.5 | 0.12 | 8 | - | - | blaACT-7 | blaTEM-1B |
| 84895 | Eclo | R | 1 | 2 | 0.5 | 2 | 32 | 0.5 | 4 | </= 0.12 | 4 | 2 | 0.5 | 8 | - | - | blaACT-15 | - |
| 84980 | Eclo | R | 0.25 | 4 | 0.5 | 4 | 1 | 0.5 | </= 0.25 | </= 0.12 | </= 0.25 | </= 0.06 | </= 0.06 | 64 | - | - | blaACT-41 | - |
| 85061 | KA | U | 0.06 | 0.06 | 0.5 | 0.5 | </= 0.25 | 0.5 | 1 | 0.5 | </= 0.25 | </= 0.06 | </= 0.06 | 4 | - | - | - | - |
| 85234 | KA | R | 0.12 | 1 | 0.5 | 8 | 1 | 0.5 | 1 | </= 0.12 | 8 | 0.25 | </= 0.06 | 64 | - | - | ampC | - |
| 85963 | Eclo | W | 0.06 | 0.12 | 2 | 4 | 2 | </= 0.25 | 2 | 0.25 | </= 0.25 | 0.25 | </= 0.06 | 32 | - | - | blaMIR-5 | - |
| 86794 | KP | R | 0.03 | </= 0.03 | </= 0.25 | 0.5 | </= 0.25 | 0.5 | </= 0.25 | 0.25 | </= 0.25 | </= 0.06 | </= 0.06 | 2 | - | - | - | blaSHV-11 |
| 87234 | Eclo | B | 0.06 | 0.25 | 1 | 2 | 0.5 | </= 0.25 | 0.5 | 0.25 | </= 0.25 | </= 0.06 | </= 0.06 | 16 | - | - | blaACT-12 | - |
| 87702 | KP | B | 0.25 | >64 | 1 | >32 | >32 | 16 | 8 | </= 0.12 | >8 | 32 | </= 0.06 | >128 | blaKPC-3 | - | - | blaOXA-9, blaSHV-11, blaTEM-1A |
| 88273 | EC | W | 1 | >64 | </= 0.25 | 1 | 4 | 1 | </= 0.25 | 0.25 | >8 | 0.5 | 0.12 | 8 | - | blaCTX-M-15 | - | blaOXA-1, blaTEM-1B |
| 88609 | EC | B | 0.06 | 0.12 | </= 0.25 | </= 0.25 | </= 0.25 | 0.5 | </= 0.25 | 0.25 | </= 0.25 | </= 0.06 | </= 0.06 | 2 | - | - | - | - |
| 88785 | Eclo | B | 0.12 | 0.25 | 0.5 | 2 | 1 | 0.5 | 2 | 0.5 | </= 0.25 | 0.25 | </= 0.06 | 16 | - | - | blaMIR-1 | - |
| 89072 | SM | B | 0.25 | 32 | 0.5 | >32 | >32 | 8 | >32 | 0.5 | 4 | 32 | </= 0.06 | >128 | blaKPC-3 | - | - | blaOXA-9, blaTEM-1C |
| 89439 | EC | B | 2 | >64 | 0.5 | 32 | 1 | >16 | </= 0.25 | </= 0.12 | >8 | 0.12 | </= 0.06 | >128 | - | blaCTX-M-15 | - | blaOXA-1 |
| 90405 | Eclo | R | 0.5 | 2 | 2 | 16 | 2 | </= 0.25 | 1 | 0.25 | </= 0.25 | 0.25 | </= 0.06 | >128 | - | - | blaACT-5 | - |
| 90693 | Eclo | W | 0.25 | 1 | 0.5 | 2 | 2 | 0.5 | 1 | 0.25 | </= 0.25 | 0.25 | </= 0.06 | 8 | - | - | blaACT-7 | - |
| 90789 | EC | R | 0.12 | 16 | 0.5 | 32 | 8 | >16 | 4 | </= 0.12 | >8 | 4 | </= 0.06 | >128 | blaKPC-3 | - | - | blaOXA-9, blaTEM-1C |
| 90942 | KO | W | 0.25 | 16 | 1 | >32 | 32 | 16 | 8 | 0.25 | >8 | 16 | </= 0.06 | >128 | blaKPC-3 | - | - | blaOXY-1-7, blaTEM-1B |
| 91191 | EC | R | 0.12 | >64 | 0.5 | 2 | 1 | >16 | </= 0.25 | 0.25 | >8 | </= 0.06 | </= 0.06 | 4 | - | blaCTX-M-14 | - | blaTEM-1B |
| 91246 | KP | B | 2 | >64 | 1 | 16 | 1 | >16 | </= 0.25 | </= 0.12 | 4 | 0.12 | </= 0.06 | >128 | - | blaCTX-M-15 | - | blaLEN16, blaOXA-1 |
| 91271 | EC | B | 0.03 | </= 0.03 | </= 0.25 | </= 0.25 | </= 0.25 | 0.5 | </= 0.25 | 0.25 | </= 0.25 | </= 0.06 | </= 0.06 | 1 | - | - | - | - |
| 91284 | Eclo | U | 0.5 | 8 | 1 | 32 | 2 | </= 0.25 | 0.5 | </= 0.12 | </= 0.25 | 0.25 | </= 0.06 | >128 | - | - | blaACT-14 | - |
| 91505 | Eclo | U | 0.25 | 4 | 1 | 1 | 2 | 0.5 | 0.5 | 0.25 | 4 | 0.25 | 0.12 | 4 | - | blaSHV-12 | blaACT-15 | - |
| 91767 | KA | R | 0.5 | 1 | 1 | >32 | 2 | 1 | 1 | 0.25 | </= 0.25 | 0.25 | 0.12 | >128 | - | - | ampC | - |
| 91871 | CF | U | 1 | 2 | 4 | >32 | 16 | >16 | 2 | 1 | >8 | 1 | 0.25 | >128 | - | - | blaCMY-110 | blaTEM-1B |
| 91996 | SM | R | 1 | 2 | 1 | 4 | 4 | 8 | 2 | 2 | 1 | 1 | 0.12 | 128 | - | - | blaSRT-2 | - |
| 92322 | Eclo | B | 0.12 | 0.25 | 0.5 | 1 | 1 | 1 | 1 | 0.25 | </= 0.25 | 0.25 | </= 0.06 | 2 | - | - | blaACT-10 | - |
| 92762 | Eclo | R | 0.5 | 2 | 2 | 4 | 1 | 2 | 0.5 | 0.25 | >8 | 0.25 | </= 0.06 | 32 | - | - | blaACT-15 | blaOXA-10 |
| 92765 | Eclo | R | 0.25 | 4 | 1 | 32 | 1 | 0.5 | </= 0.25 | </= 0.12 | </= 0.25 | 0.25 | </= 0.06 | 128 | - | - | blaACT-7 | - |
| 92862 | KA | B | 0.12 | 0.5 | 0.5 | 8 | 1 | 0.5 | 2 | 0.5 | 0.5 | 0.25 | </= 0.06 | 128 | - | - | ampC | - |
| 92969 | EC | U | 2 | >64 | 0.5 | 1 | 4 | >16 | </= 0.25 | 0.25 | 8 | 0.25 | </= 0.06 | >128 | - | blaCTX-M-15 | - | blaOXA-1 |
| 92980 | MM | U | 0.25 | 0.25 | 0.5 | 2 | 2 | </= 0.25 | 4 | 2 | 0.5 | 0.5 | 0.12 | 1 | - | - | blaDHA-12 | - |
| 93209 | Eclo | R | 0.06 | 0.12 | </= 0.25 | 0.5 | </= 0.25 | 0.5 | 1 | 0.25 | </= 0.25 | </= 0.06 | </= 0.06 | 2 | - | - | blaACT-7 | - |
| 93220 | Eclo | R | 0.06 | </= 0.03 | </= 0.25 | </= 0.25 | </= 0.25 | </= 0.25 | 1 | 0.25 | </= 0.25 | </= 0.06 | </= 0.06 | 2 | - | - | blaMIR-2 | - |
| 93869 | Eclo | B | 1 | 4 | 2 | 32 | 2 | 1 | 1 | 1 | 2 | 0.25 | 0.12 | 128 | - | - | blaACT-7 | - |
| 93871 | Eclo | B | 1 | 2 | 2 | 16 | 2 | 2 | 0.5 | 0.25 | 2 | 0.25 | 0.12 | >128 | - | - | blaACT-7 | - |
| 93960 | EC | B | 0.25 | 4 | 1 | 32 | 1 | 8 | 1 | 0.25 | >8 | 0.25 | </= 0.06 | 128 | - | - | blaCMY-2 | - |
| 94179 | Eclo | B | 0.12 | 0.12 | 0.5 | 0.5 | </= 0.25 | </= 0.25 | </= 0.25 | 0.25 | </= 0.25 | </= 0.06 | </= 0.06 | 2 | - | - | blaACT-5 | - |
| 94353 | KA | B | 0.03 | </= 0.03 | </= 0.25 | </= 0.25 | </= 0.25 | 0.5 | 0.5 | 0.5 | </= 0.25 | </= 0.06 | </= 0.06 | 1 | - | - | - | blaSHV-1 |
| 94620 | Eclo | R | 0.25 | 1 | 2 | 8 | 4 | </= 0.25 | 1 | 0.25 | </= 0.25 | 0.5 | </= 0.06 | 64 | - | - | blaMIR-2 | - |
| 94720 | Eclo | B | 2 | 8 | 1 | 32 | 8 | </= 0.25 | 0.5 | </= 0.12 | 0.5 | 0.25 | 0.12 | >128 | - | - | blaACT-7 | - |
| 95009 | KA | B | 2 | 2 | 2 | 32 | 16 | 2 | 8 | 1 | >8 | 0.5 | 0.25 | 128 | - | - | ampC | - |
| 95404 | KA | W | 1 | 2 | 1 | 1 | 32 | 0.5 | 16 | 1 | 4 | 8 | 2 | 8 | - | - | ampC | - |
| 95556 | Eclo | B | 0.5 | 4 | 1 | 16 | 1 | 0.5 | 0.5 | 0.25 | </= 0.25 | 0.5 | </= 0.06 | >128 | - | - | blaACT-15 | - |
| 95575 | Eclo | U | 0.25 | 1 | 1 | 1 | 2 | >16 | 2 | 0.5 | 2 | 1 | 0.12 | 4 | - | blaSHV-12 | blaACT-7 | blaTEM-1B |
| 95576 | Eclo | U | 0.25 | 4 | 1 | 1 | 1 | >16 | 0.5 | 0.25 | 2 | 0.12 | </= 0.06 | 8 | - | blaSHV-12 | blaACT-7 | blaTEM-1B |
| 95648 | Eclo | R | 1 | 16 | 0.5 | 16 | 2 | >16 | </= 0.25 | </= 0.12 | >8 | </= 0.06 | </= 0.06 | >128 | - | blaSHV-12 | blaACT-7 | blaTEM-1B |
| 95712 | Eclo | R | 1 | 16 | 1 | 16 | 2 | >16 | 0.5 | </= 0.12 | >8 | 0.12 | </= 0.06 | >128 | - | blaSHV-12 | blaACT-7 | blaTEM-1B |
| 95882 | EC | R | 0.12 | >64 | 2 | 32 | 16 | 2 | 4 | 1 | >8 | 8 | </= 0.06 | >128 | blaKPC-3 | - | - | blaTEM-1C |
| 95959 | Eclo | W | 2 | 4 | 4 | 16 | 8 | 0.5 | 1 | 0.5 | 8 | 0.5 | 0.12 | >128 | - | - | blaACT-16 | - |
| 95999 | KA | R | 0.06 | 0.25 | 0.5 | 2 | 1 | 0.5 | 1 | 0.5 | 0.5 | </= 0.06 | </= 0.06 | 16 | - | - | ampC | - |
| 96129 | KA | R | 0.25 | 1 | 0.5 | 16 | 1 | 1 | 0.5 | </= 0.12 | 0.5 | 0.12 | </= 0.06 | >128 | - | - | ampC | - |
| 96415 | Eclo | R | 0.06 | </= 0.03 | </= 0.25 | </= 0.25 | </= 0.25 | </= 0.25 | </= 0.25 | 0.25 | </= 0.25 | </= 0.06 | </= 0.06 | 2 | - | - | - | - |
| 96678 | Eclo | R | 0.25 | 32 | 1 | 4 | 0.5 | 1 | 2 | 0.5 | 1 | 0.5 | 0.12 | 128 | - | blaSHV-12 | blaACT-3 | blaTEM-1B |
| 96924 | Eclo | R | 0.5 | 4 | 1 | 32 | 1 | </= 0.25 | 0.5 | 0.25 | </= 0.25 | 0.12 | </= 0.06 | 128 | - | - | blaACT-7 | - |
| 97085 | Eclo | R | 0.06 | 0.06 | </= 0.25 | 0.5 | </= 0.25 | 0.5 | 1 | 0.25 | </= 0.25 | </= 0.06 | </= 0.06 | 2 | - | - | - | - |
| 97104 | Eclo | R | 0.25 | 0.25 | 0.5 | 0.5 | </= 0.25 | 0.5 | </= 0.25 | 0.25 | </= 0.25 | </= 0.06 | </= 0.06 | 4 | - | - | blaACT-15 | - |
| 97115 | Eclo | R | 0.25 | 4 | 1 | 16 | 1 | 0.5 | 0.5 | 0.25 | </= 0.25 | 0.5 | </= 0.06 | 128 | - | - | blaACT-15 | - |
| 97239 | EC | B | 0.06 | </= 0.03 | </= 0.25 | </= 0.25 | </= 0.25 | </= 0.25 | </= 0.25 | 0.25 | </= 0.25 | </= 0.06 | </= 0.06 | 1 | - | - | - | blaTEM-1C |
| 97571 | Eclo | R | 0.25 | 0.5 | 1 | 8 | 1 | 0.5 | 1 | 0.25 | </= 0.25 | 0.12 | </= 0.06 | 32 | - | - | blaACT-9 | - |
| 98131 | KP | B | 0.25 | 0.5 | </= 0.25 | 0.5 | 1 | </= 0.25 | 0.5 | 0.25 | 1 | 0.25 | 0.12 | 4 | - | - | - | blaSHV-108 |
| 98374 | SM | B | 1 | 1 | 2 | 2 | 1 | 4 | 2 | 2 | </= 0.25 | 0.12 | </= 0.06 | 32 | - | - | blaSRT-2 | - |
| 98545 | KA | U | 0.25 | 0.5 | </= 0.25 | 2 | 1 | 0.5 | 1 | 0.5 | 0.5 | 0.12 | </= 0.06 | 32 | - | - | ampC | - |
| 98550 | EC | U | 0.5 | >64 | 0.5 | 4 | 2 | 2 | </= 0.25 | </= 0.12 | >8 | 0.12 | </= 0.06 | 32 | - | blaCTX-M-15 | - | blaOXA-1 |
| 98787 | Eclo | R | 0.5 | 4 | 0.5 | 16 | 1 | 0.5 | 0.5 | 0.25 | </= 0.25 | 0.12 | </= 0.06 | 128 | - | - | blaACT-14 | - |
| 98793 | Eclo | R | 0.12 | 4 | 8 | 32 | 2 | 8 | 2 | 0.5 | 0.5 | 0.5 | </= 0.06 | >128 | - | - | blaACT-7, blaFOX-5 | blaCARB-2 |
| 99019 | Eclo | B | 0.06 | 0.06 | 0.5 | 0.5 | </= 0.25 | </= 0.25 | </= 0.25 | 0.25 | </= 0.25 | </= 0.06 | </= 0.06 | 4 | - | - | blaACT-5 | - |
| 99085 | Eclo | B | 0.25 | 4 | 0.5 | 16 | 1 | 0.5 | </= 0.25 | </= 0.12 | </= 0.25 | 0.12 | </= 0.06 | 128 | - | - | blaACT-5 | - |
| 99436 | Eclo | B | 0.25 | 2 | 1 | 8 | 1 | 0.5 | </= 0.25 | 0.25 | </= 0.25 | 0.12 | </= 0.06 | 64 | - | - | blaACT-16 | - |
| 100546 | Eclo | B | 0.5 | 4 | 1 | 16 | 1 | </= 0.25 | </= 0.25 | </= 0.12 | </= 0.25 | 0.12 | 0.12 | 128 | - | - | blaACT-7 | - |
| 100700 | KA | R | 0.12 | 2 | 0.5 | 1 | 0.5 | 2 | 2 | 0.5 | </= 0.25 | </= 0.06 | </= 0.06 | 4 | - | - | ampC | - |
| 101026 | Eclo | R | 0.06 | 0.06 | </= 0.25 | </= 0.25 | </= 0.25 | 0.5 | 0.5 | 0.25 | </= 0.25 | </= 0.06 | </= 0.06 | 2 | - | - | blaACT-7 | - |
| 101211 | Eclo | R | 0.5 | 4 | </= 0.25 | 8 | 2 | </= 0.25 | 0.5 | </= 0.12 | >8 | 0.12 | 0.12 | 64 | - | - | blaACT-35 | - |
| 101616 | Eclo | R | 0.25 | 2 | 1 | 8 | 1 | 0.5 | </= 0.25 | 0.25 | </= 0.25 | 0.12 | </= 0.06 | >128 | - | - | blaACT-5 | - |
| 101683 | KP | B | 1 | 64 | 32 | 32 | 32 | 0.5 | 2 | 0.5 | >8 | 4 | 4 | >128 | blaNDM-1, blaOXA-232 | blaCTX-M-15 | - | blaOXA-9, blaSHV-1, blaTEM-1C |
| 101753 | Eclo | B | 1 | 1 | 1 | 1 | 2 | 1 | 8 | 2 | </= 0.25 | 0.5 | 0.12 | 8 | - | - | blaMIR-3 | - |
| 101812 | Eclo | R | 0.25 | 8 | 4 | 16 | 1 | 0.5 | </= 0.25 | 0.25 | </= 0.25 | 0.25 | </= 0.06 | >128 | - | - | blaACT-15 | - |
| 101867 | Eclo | B | 0.5 | 4 | 0.5 | 8 | 32 | </= 0.25 | 4 | 0.25 | 4 | 4 | 0.5 | 32 | - | - | blaACT-14 | - |
| 101910 | Eclo | B | 0.12 | 32 | 2 | >32 | 1 | </= 0.25 | 0.5 | 0.25 | 2 | 0.12 | </= 0.06 | >128 | - | blaSHV-12 | blaACT-15 | blaTEM-1B |
| 101913 | Eclo | B | 1 | 4 | 1 | 16 | 1 | </= 0.25 | 0.5 | </= 0.12 | </= 0.25 | 0.25 | </= 0.06 | 128 | - | - | blaACT-7 | - |
| 101990 | KA | R | 0.12 | 0.5 | 0.5 | 8 | 1 | 0.5 | 1 | 0.25 | 1 | 0.12 | </= 0.06 | 64 | - | - | ampC | - |
| 102065 | Eclo | B | 0.25 | 4 | 1 | 8 | 1 | </= 0.25 | </= 0.25 | 0.25 | </= 0.25 | </= 0.06 | </= 0.06 | 128 | - | - | blaACT-15 | - |
| 102338 | Eclo | B | 0.5 | 8 | 2 | 32 | 1 | 0.5 | 0.5 | 0.25 | </= 0.25 | 0.25 | </= 0.06 | >128 | - | - | blaACT-15 | - |
| 103137 | KP | B | 0.06 | 0.25 | </= 0.25 | >32 | 1 | 0.5 | 1 | </= 0.12 | 0.5 | 0.12 | </= 0.06 | >128 | - | - | blaDHA-1 | blaLEN25 |
| 103359 | Eclo | R | 0.06 | 0.12 | </= 0.25 | 0.5 | </= 0.25 | </= 0.25 | 1 | 0.25 | </= 0.25 | </= 0.06 | </= 0.06 | 2 | - | - | blaACT-7 | - |
| 103617 | Eclo | U | 0.25 | 1 | 0.5 | 4 | 4 | </= 0.25 | 1 | 0.25 | 4 | 0.5 | </= 0.06 | 32 | - | - | blaMIR-6 | - |
| 104067 | Eclo | R | 4 | 8 | 8 | >32 | >32 | </= 0.25 | 32 | 4 | 0.5 | 16 | 8 | 128 | - | - | blaACT-7 | - |
| 104071 | KA | R | 0.12 | 0.5 | 1 | 16 | 1 | 0.5 | 1 | 1 | </= 0.25 | 0.12 | </= 0.06 | >128 | - | - | ampC | - |
| 104350 | EC | R | 0.12 | 0.25 | </= 0.25 | 32 | 1 | >16 | 1 | </= 0.12 | >8 | </= 0.06 | </= 0.06 | 64 | - | - | blaDHA-1 | - |
| 104422 | KA | R | 0.06 | 0.06 | </= 0.25 | 0.5 | </= 0.25 | </= 0.25 | 1 | 0.5 | </= 0.25 | </= 0.06 | </= 0.06 | 4 | - | - | - | - |
| 104904 | Eclo | R | 0.25 | 2 | 1 | 8 | 1 | 0.5 | 0.5 | 0.25 | </= 0.25 | 0.25 | 0.12 | 128 | - | - | blaACT-7 | - |
| 106256 | KP | B | 4 | >64 | 2 | >32 | 16 | >16 | 1 | 0.25 | >8 | 2 | 0.5 | >128 | - | blaCTX-M-15 | - | blaOXA-1, blaOXA-9, blaSHV-28, blaTEM-1A |
| 106698 | KP | B | 8 | >64 | 4 | >32 | 8 | >16 | 0.5 | 0.5 | >8 | 2 | 0.5 | >128 | - | blaCTX-M-15 | - | blaOXA-1, blaOXA-9, blaSHV-28, blaTEM-1A |
| 107019 | SM | B | 0.5 | 2 | 1 | 2 | 1 | </= 0.25 | 1 | 1 | >8 | 0.12 | 0.12 | 32 | - | - | blaSRT-2 | - |
| 107115 | EC | R | 32 | >64 | >32 | >32 | >32 | >16 | 8 | 4 | >8 | 32 | 16 | >128 | - | blaCTX-M-71 | - | - |
| 107154 | EC | B | 0.25 | 2 | 0.5 | 8 | 2 | 0.5 | 1 | 0.25 | </= 0.25 | 0.25 | 0.12 | 64 | - | - | - | blaTEM-1B |
| 107336 | Eclo | B | 0.12 | 4 | 0.5 | 1 | 0.5 | 16 | 0.5 | 0.25 | 8 | 0.12 | </= 0.06 | 4 | - | blaCTX-M-9 | blaACT-16 | - |
| 107697 | Eclo | R | 0.12 | 0.25 | 1 | 4 | 1 | 0.5 | 1 | 0.25 | </= 0.25 | 0.25 | </= 0.06 | 64 | - | - | blaACT-9 | - |
| 108225 | EC | R | 0.25 | >64 | 0.5 | 2 | 2 | >16 | </= 0.25 | </= 0.12 | >8 | 0.12 | </= 0.06 | 8 | - | blaCTX-M-15 | - | - |
| 108241 | SM | R | 0.5 | 0.5 | 0.5 | 1 | >32 | 1 | >32 | 32 | 1 | >32 | 0.12 | 8 | blaSME-3 | - | blaSRT-2 | - |
| 108883 | Eclo | W | 0.25 | 1 | 1 | 16 | 2 | 0.5 | 0.5 | 0.25 | </= 0.25 | 0.25 | </= 0.06 | 64 | - | - | blaACT-7 | - |
| 109012 | KA | R | 0.5 | 1 | 0.5 | 4 | 2 | 0.5 | 2 | 0.25 | 0.5 | 0.5 | </= 0.06 | 64 | - | - | ampC | - |
| 109431 | Eclo | R | 0.25 | 0.5 | 0.5 | 4 | 1 | 0.5 | 0.5 | </= 0.12 | >8 | 0.25 | </= 0.06 | 64 | - | - | blaACT-5, blaDHA-1 | blaOXA-1 |
| 110036 | KP | W | 4 | >64 | 2 | >32 | >32 | >16 | 4 | 4 | >8 | 16 | 8 | >128 | blaOXA-48 | blaCTX-M-15 | - | blaOXA-1, blaSCO-1, blaSHV-1, blaTEM-1A |
| 110113 | Eclo | R | 1 | 4 | 0.5 | 32 | 4 | 0.5 | 0.5 | </= 0.12 | 0.5 | 0.12 | </= 0.06 | >128 | - | - | blaACT-15 | blaTEM-1B |
| 110352 | Eclo | R | 0.25 | 8 | 1 | 16 | 1 | 0.5 | 0.5 | 0.25 | </= 0.25 | 0.12 | </= 0.06 | >128 | - | - | blaACT-15 | - |
| 110708 | Eclo | R | 0.25 | 4 | 1 | 16 | 1 | 0.5 | 0.5 | </= 0.12 | </= 0.25 | 0.12 | 0.12 | >128 | - | - | blaACT-7 | - |
| 110984 | Eclo | B | 0.25 | 1 | 1 | 16 | 2 | </= 0.25 | 1 | 0.25 | </= 0.25 | 0.25 | </= 0.06 | 128 | - | - | blaACT-9 | - |
| 111757 | KP | B | 4 | >64 | 2 | >32 | 2 | >16 | 1 | 1 | 1 | 0.25 | 0.12 | >128 | - | blaCTX-M-15 | - | blaOXA-1, blaSHV-11, blaTEM-1B |
| 111864 | KP | B | 0.03 | </= 0.03 | </= 0.25 | </= 0.25 | </= 0.25 | >16 | </= 0.25 | 0.25 | </= 0.25 | </= 0.06 | </= 0.06 | 4 | - | - | - | blaSHV-65 |
| 111952 | EC | B | 0.5 | >64 | 1 | >32 | 8 | >16 | 1 | 0.5 | </= 0.25 | 0.5 | 0.25 | >128 | - | blaCTX-M-15 | - | - |
| 113974 | EC | B | 0.03 | </= 0.03 | </= 0.25 | </= 0.25 | </= 0.25 | 0.5 | </= 0.25 | 0.25 | </= 0.25 | </= 0.06 | </= 0.06 | 1 | - | - | - | - |
| 114298 | KP | B | 1 | >64 | 1 | 8 | 1 | >16 | 0.5 | </= 0.12 | >8 | </= 0.06 | </= 0.06 | >128 | - | blaCTX-M-15 | blaDHA-1 | blaOXA-1, blaSHV-11, blaTEM-34 |
| 114336 | Eclo | R | 1 | 1 | 0.5 | 16 | 2 | >16 | 0.5 | </= 0.12 | >8 | 0.12 | </= 0.06 | 128 | - | - | blaACT-7, blaDHA-1 | blaTEM-1B |
| 114640 | KP | R | 0.03 | 0.06 | </= 0.25 | 1 | </= 0.25 | </= 0.25 | </= 0.25 | 0.25 | </= 0.25 | </= 0.06 | </= 0.06 | 8 | - | - | - | blaSHV-11 |
| 114729 | Eclo | R | 1 | 8 | 1 | 32 | 8 | 0.5 | 0.5 | </= 0.12 | </= 0.25 | 0.25 | 0.12 | >128 | - | - | blaACT-7 | - |
| 114768 | KP | B | 8 | >64 | 2 | >32 | >32 | 0.5 | 4 | 2 | 8 | 8 | 4 | >128 | - | blaCTX-M-15 | - | blaOXA-1, blaSHV-1, blaTEM-1B |
| 114870 | KP | B | 1 | >64 | >32 | >32 | 32 | >16 | 16 | 16 | >8 | >32 | >32 | >128 | blaNDM-1, blaOXA-232 | blaCTX-M-15 | - | blaOXA-9, blaSHV-1, blaTEM-1C |
| 115515 | EC | R | 0.25 | 2 | 1 | 8 | 2 | 0.5 | 1 | 0.5 | </= 0.25 | 0.12 | </= 0.06 | 128 | - | - | blaCMY-2 | - |
| 115528 | Eclo | R | 0.5 | 8 | 1 | 16 | 2 | 0.5 | </= 0.25 | </= 0.12 | </= 0.25 | 0.12 | </= 0.06 | >128 | - | - | blaACT-7 | - |
| 115835 | Eclo | B | 1 | 2 | 2 | 8 | 2 | 0.5 | 1 | </= 0.12 | 8 | 0.5 | 0.12 | 128 | - | - | blaCMH-3 | blaOXA-1 |
| 115869 | Eclo | B | 0.25 | 0.25 | 0.5 | 0.5 | 2 | 0.5 | 0.5 | </= 0.12 | </= 0.25 | 0.25 | </= 0.06 | 2 | - | - | blaACT-7 | - |
| 116948 | Eclo | R | 1 | 8 | 1 | 32 | 16 | </= 0.25 | 2 | 0.25 | </= 0.25 | 2 | 0.5 | 128 | - | - | blaACT-7 | - |
| 116959 | Eclo | R | 1 | 8 | 1 | 32 | 16 | 0.5 | 2 | 0.25 | 0.5 | 4 | 0.5 | >128 | - | - | blaACT-7 | - |
| 117039 | Eclo | B | 0.06 | 0.5 | 0.5 | 2 | 0.5 | </= 0.25 | 0.5 | 0.5 | </= 0.25 | 0.12 | </= 0.06 | 16 | - | - | blaACT-12 | - |
| 117868 | KP | W | 0.5 | >64 | 2 | >32 | 2 | >16 | 1 | 0.5 | >8 | 0.12 | </= 0.06 | >128 | - | blaCTX-M-15 | blaDHA-1 | blaOXA-1, blaSHV-11, blaTEM-1B |
| 117914 | Eclo | R | 0.06 | 0.12 | </= 0.25 | 0.5 | </= 0.25 | </= 0.25 | 0.5 | 0.25 | </= 0.25 | </= 0.06 | </= 0.06 | 2 | - | - | blaACT-15 | blaTEM-1B |
| 118015 | Eclo | B | 0.06 | 0.12 | </= 0.25 | 0.5 | </= 0.25 | </= 0.25 | 0.5 | 0.25 | </= 0.25 | </= 0.06 | </= 0.06 | 2 | - | - | blaACT-7 | - |
| 118283 | Eclo | R | 2 | 8 | 2 | 16 | 1 | </= 0.25 | </= 0.25 | </= 0.12 | 4 | </= 0.06 | </= 0.06 | >128 | - | - | blaACT-16 | - |
| 118315 | Eclo | R | 1 | 4 | 1 | 16 | 1 | </= 0.25 | </= 0.25 | </= 0.12 | </= 0.25 | 0.12 | </= 0.06 | >128 | - | - | blaACT-15 | - |
| 118350 | Eclo | R | 0.25 | 1 | 0.5 | 8 | 0.5 | 1 | 0.5 | </= 0.12 | </= 0.25 | </= 0.06 | </= 0.06 | 32 | - | - | blaACT-14 | - |
| 119178 | KP | B | 1 | >64 | 1 | >32 | 8 | >16 | 2 | 0.25 | >8 | 0.5 | </= 0.06 | >128 | - | blaCTX-M-15 | blaDHA-1 | blaOXA-1, blaSHV-11, blaTEM-1B |
| 119464 | KP | B | 1 | >64 | 8 | >32 | 8 | 0.5 | 2 | 0.5 | 1 | 2 | 2 | >128 | - | - | - | blaTEM-1B |
| 119589 | Eclo | B | 0.12 | 2 | 0.5 | 4 | 0.5 | 0.5 | 0.5 | 0.25 | </= 0.25 | 0.12 | </= 0.06 | 64 | - | - | blaACT-16 | - |
| 119972 | Eclo | B | 0.5 | 0.5 | 0.5 | 1 | 2 | >16 | 0.5 | 0.25 | >8 | 0.12 | </= 0.06 | 32 | - | - | blaACT-5 | - |
| 120040 | Eclo | B | 0.5 | >64 | 8 | >32 | 4 | 0.5 | 2 | 1 | >8 | 0.25 | </= 0.06 | >128 | - | - | blaACT-15 | blaTEM-1B |
| 121023 | Eclo | B | 0.25 | 1 | 1 | 16 | 2 | </= 0.25 | 1 | 0.25 | </= 0.25 | 0.5 | </= 0.06 | 128 | - | - | blaACT-7 | - |
| 121966 | CF | W | 0.25 | 4 | 1 | >32 | 2 | 16 | 0.5 | 0.25 | 1 | 0.25 | </= 0.06 | >128 | - | - | blaCMY-78 | blaTEM-1B |
| 122215 | KP | R | 4 | >64 | 1 | >32 | 1 | 0.5 | 1 | </= 0.12 | >8 | 0.12 | </= 0.06 | >128 | - | blaCTX-M-15 | blaDHA-1 | blaOXA-1, blaOXA-10, blaSHV-11, blaTEM-1B |
| 122288 | KA | R | 0.06 | 0.25 | </= 0.25 | 4 | 1 | </= 0.25 | 1 | </= 0.12 | >8 | 0.12 | </= 0.06 | 32 | - | - | ampC | - |
| 122295 | Eclo | R | 0.25 | 1 | 1 | 16 | 2 | </= 0.25 | 1 | 0.25 | </= 0.25 | 0.25 | </= 0.06 | 64 | - | - | blaACT-49 | - |
| 122303 | SM | R | 4 | 8 | 4 | 8 | 16 | 2 | 4 | 2 | 8 | 4 | 2 | 64 | - | - | blaSST-1 | - |
| 122323 | SM | R | 2 | 2 | 4 | 4 | 0.5 | 4 | 1 | 1 | 0.5 | </= 0.06 | </= 0.06 | 4 | - | - | - | - |
| 122511 | Eclo | R | 2 | 8 | 4 | 16 | 4 | </= 0.25 | 0.5 | 0.25 | 2 | 0.5 | </= 0.06 | >128 | - | - | blaACT-7 | - |
| 122635 | KP | U | 0.12 | 0.12 | </= 0.25 | 0.5 | 0.5 | </= 0.25 | </= 0.25 | 0.25 | 1 | </= 0.06 | </= 0.06 | 4 | - | blaSHV-38 | - | - |
| 122658 | Eclo | R | 0.06 | 0.25 | 1 | 2 | 1 | 0.5 | 1 | 0.25 | </= 0.25 | 0.25 | </= 0.06 | 32 | - | - | blaMIR-6 | - |
| 122663 | KP | R | 0.03 | 0.06 | </= 0.25 | </= 0.25 | </= 0.25 | </= 0.25 | </= 0.25 | 0.5 | </= 0.25 | </= 0.06 | </= 0.06 | 4 | - | - | - | blaSHV-36 |
| 123075 | Eclo | R | 0.25 | 0.5 | 1 | 2 | 0.5 | 0.5 | 0.5 | 0.25 | 4 | 0.12 | </= 0.06 | 16 | - | - | blaACT-7 | - |
| 123238 | KP | U | 0.12 | 32 | </= 0.25 | 8 | 1 | </= 0.25 | 1 | 0.25 | >8 | 0.25 | 0.12 | 128 | blaOXA-181 | blaCTX-M-15 | - | blaOXA-1, blaTEM-1B |
| 123274 | Eclo | R | 0.25 | 4 | 1 | 16 | 1 | 0.5 | </= 0.25 | 0.25 | </= 0.25 | 0.12 | </= 0.06 | 128 | - | - | blaACT-15 | - |
| 123310 | Eclo | R | 0.5 | 8 | 1 | 16 | 1 | 0.5 | </= 0.25 | 0.25 | >8 | 0.25 | </= 0.06 | 128 | - | - | blaACT-15 | - |
| 123338 | Eclo | R | 0.5 | 4 | 1 | 8 | 1 | 0.5 | </= 0.25 | 0.25 | </= 0.25 | 0.12 | </= 0.06 | 128 | - | - | blaACT-15 | - |
| 123376 | EC | B | 0.12 | 1 | 0.5 | 8 | 0.5 | 0.5 | 0.5 | 0.25 | >8 | </= 0.06 | </= 0.06 | 32 | - | - | blaCMY-42 | - |
| 123515 | SM | R | 0.5 | 0.5 | 0.5 | 1 | 0.5 | </= 0.25 | 1 | 0.5 | 1 | </= 0.06 | </= 0.06 | 8 | - | - | blaSRT-2 | - |
| 123547 | PM | B | 0.06 | 0.25 | </= 0.25 | 0.5 | 0.5 | 16 | 4 | 4 | </= 0.25 | 0.12 | 0.12 | </= 0.5 | - | - | - | - |
| 123814 | Eclo | U | 0.25 | 2 | 0.5 | 8 | 1 | </= 0.25 | </= 0.25 | 0.25 | </= 0.25 | 0.12 | </= 0.06 | 64 | - | - | blaACT-14 | - |
| 123888 | Eclo | R | 0.25 | 0.5 | 1 | 2 | 1 | 0.5 | 1 | 0.25 | </= 0.25 | 0.25 | </= 0.06 | 32 | - | - | blaACT-50 | - |
| 124086 | Eclo | R | 0.06 | 0.12 | </= 0.25 | 0.5 | </= 0.25 | 0.5 | 0.5 | 0.25 | </= 0.25 | </= 0.06 | </= 0.06 | 2 | - | - | blaACT-7 | - |
| 124114 | Eclo | R | 0.06 | 0.25 | 0.5 | 2 | 0.5 | 1 | 0.5 | 0.25 | 4 | </= 0.06 | </= 0.06 | 32 | - | - | blaMIR-3 | - |
| 124136 | Eclo | R | 0.12 | </= 0.03 | </= 0.25 | 0.5 | </= 0.25 | 0.5 | 1 | 0.25 | </= 0.25 | </= 0.06 | </= 0.06 | 4 | - | - | blaACT-2 | - |
| 124218 | Eclo | U | 0.25 | 4 | 0.5 | 8 | 1 | 0.5 | </= 0.25 | </= 0.12 | </= 0.25 | </= 0.06 | </= 0.06 | 128 | - | - | blaACT-14 | - |
| 124258 | Eclo | R | 4 | 32 | 8 | >32 | 16 | >16 | 4 | 0.5 | 8 | 2 | 1 | >128 | - | blaSHV-12 | blaACT-15 | - |
| 124415 | Eclo | R | 1 | 2 | 1 | 16 | 2 | </= 0.25 | </= 0.25 | </= 0.12 | </= 0.25 | 0.12 | </= 0.06 | 128 | - | - | blaACT-7 | - |
| 124439 | Eclo | R | 0.5 | 4 | 0.5 | 8 | 2 | 0.5 | </= 0.25 | </= 0.12 | </= 0.25 | 0.25 | </= 0.06 | 64 | - | - | blaACT-7 | - |
| 124447 | Eclo | R | 0.12 | 2 | 0.5 | 16 | 0.5 | 0.5 | 0.5 | 0.25 | </= 0.25 | 0.12 | </= 0.06 | >128 | - | - | blaACT-7 | - |
| 124824 | EC | B | 0.5 | 8 | 1 | >32 | 2 | >16 | 1 | 0.25 | 0.5 | 0.25 | </= 0.06 | >128 | - | - | blaCMY-2 | blaTEM-1C |
| 124860 | EC | B | 0.06 | 8 | 0.5 | 0.5 | 4 | 1 | </= 0.25 | 0.25 | >8 | 1 | 0.12 | 4 | - | blaCTX-M-67 | - | - |
| 125629 | KA | W | 2 | 16 | 4 | 32 | >32 | 0.5 | 32 | 8 | 0.5 | 32 | 16 | >128 | - | - | ampC | - |
| 125677 | EC | B | 1 | >64 | 0.5 | 1 | 1 | >16 | </= 0.25 | 0.25 | >8 | </= 0.06 | </= 0.06 | 32 | - | blaCTX-M-15 | - | blaOXA-1 |
| 125696 | KA | B | 0.12 | 0.5 | 0.5 | 2 | 2 | 0.5 | 1 | </= 0.12 | 1 | 0.25 | </= 0.06 | 32 | - | - | ampC | - |
| 125709 | KP | B | 4 | >64 | 0.5 | 2 | 8 | >16 | </= 0.25 | 0.25 | >8 | 1 | 0.5 | 32 | - | blaCTX-M-15 | - | blaOXA-1, blaSHV-11, blaTEM-1B |
| 125801 | Eclo | B | 0.25 | >64 | 4 | >32 | 2 | 0.5 | 0.5 | 0.25 | </= 0.25 | 0.25 | </= 0.06 | >128 | - | - | blaACT-12 | blaSFO-1 |
| 125997 | KA | R | 0.12 | 0.5 | 1 | 8 | 1 | 1 | 1 | 0.25 | </= 0.25 | 0.12 | </= 0.06 | 128 | - | - | ampC | - |
| 126518 | EC | U | 0.5 | 16 | 0.5 | 1 | 1 | 1 | </= 0.25 | </= 0.12 | >8 | 0.25 | 0.25 | 4 | - | blaCTX-M-15 | - | blaOXA-1 |
| 126524 | Eclo | U | 0.25 | 0.5 | 0.5 | 4 | 1 | </= 0.25 | 0.5 | 0.25 | </= 0.25 | 0.25 | </= 0.06 | 32 | - | - | blaACT-9 | - |
| 126564 | Eclo | R | 0.25 | 1 | 1 | 4 | 1 | 0.5 | 0.5 | </= 0.12 | </= 0.25 | 0.12 | </= 0.06 | 64 | - | - | blaACT-12 | - |
| 126769 | EC | R | 0.25 | 0.25 | </= 0.25 | </= 0.25 | 1 | 0.5 | </= 0.25 | </= 0.12 | </= 0.25 | 0.25 | 0.12 | 4 | - | - | blaEC-18 | - |
| 127012 | Eclo | R | 0.5 | 2 | 2 | 32 | 1 | </= 0.25 | 0.5 | 0.25 | </= 0.25 | 0.12 | </= 0.06 | >128 | - | - | blaACT-7 | - |
| 127189 | KA | B | 0.06 | 0.25 | 0.5 | 4 | 1 | 0.5 | 1 | </= 0.12 | </= 0.25 | 0.12 | </= 0.06 | 64 | - | - | ampC | - |
| 127252 | Eclo | U | 0.12 | 1 | 2 | 4 | 1 | 0.5 | </= 0.25 | 0.25 | </= 0.25 | </= 0.06 | </= 0.06 | 32 | - | - | blaACT-7 | - |
| 127300 | KA | R | 1 | 2 | 0.5 | 8 | 4 | </= 0.25 | 1 | </= 0.12 | 1 | 0.5 | 0.12 | 64 | - | - | ampC | - |
| 127330 | Eclo | R | 0.03 | </= 0.03 | </= 0.25 | </= 0.25 | </= 0.25 | 0.5 | 1 | 0.5 | </= 0.25 | </= 0.06 | </= 0.06 | 2 | - | - | - | - |
| 127393 | SM | W | 0.5 | 0.5 | 1 | 2 | 1 | 2 | 2 | 1 | </= 0.25 | 0.12 | </= 0.06 | 64 | - | - | blaSRT-1 | - |
| 127466 | KA | R | 0.06 | 0.25 | 0.5 | 8 | 0.5 | 0.5 | 0.5 | 0.25 | </= 0.25 | </= 0.06 | </= 0.06 | 64 | - | - | ampC | - |
| 127902 | Eclo | R | 0.5 | 0.5 | 1 | 2 | 1 | 1 | 1 | 1 | 1 | 0.12 | </= 0.06 | 16 | - | - | blaACT-49 | - |
| 128143 | KP | R | 1 | >64 | 2 | >32 | 1 | >16 | 2 | 1 | >8 | 0.25 | </= 0.06 | >128 | - | blaCTX-M-15 | blaDHA-1 | blaOXA-1, blaSHV-11, blaTEM-1B |
| 128219 | Eclo | B | 0.5 | 2 | 1 | 16 | 2 | </= 0.25 | 0.5 | 0.25 | </= 0.25 | 0.25 | </= 0.06 | >128 | - | - | blaACT-7 | - |
| 128308 | Eclo | R | 2 | 8 | 2 | >32 | 2 | </= 0.25 | </= 0.25 | </= 0.12 | 4 | 0.12 | </= 0.06 | >128 | - | - | blaACT-15 | - |
| 128339 | KP | B | 1 | >64 | 2 | 16 | 32 | </= 0.25 | 16 | 1 | >8 | 16 | 2 | >128 | - | blaCTX-M-3, blaSHV-27 | - | blaTEM-1B |
| 128553 | Eclo | B | 0.5 | 4 | 1 | 8 | 1 | </= 0.25 | </= 0.25 | </= 0.12 | </= 0.25 | </= 0.06 | </= 0.06 | 128 | - | - | blaACT-5 | - |
| 128623 | KP | R | 0.5 | 8 | 1 | 32 | 16 | </= 0.25 | 8 | 0.5 | 8 | 8 | </= 0.06 | >128 | blaKPC-2 | - | - | blaSHV-11, blaTEM-1B |
| 128626 | Eclo | R | 0.25 | 4 | 2 | 16 | 2 | 0.5 | 0.5 | 0.25 | 0.5 | 0.25 | </= 0.06 | 128 | - | - | blaACT-7 | - |
| 129044 | Eclo | R | 0.25 | 4 | 0.5 | 16 | 1 | 0.5 | </= 0.25 | </= 0.12 | </= 0.25 | 0.12 | </= 0.06 | 64 | - | - | blaACT-15 | - |
| 129117 | EC | R | 2 | 32 | </= 0.25 | >32 | 2 | </= 0.25 | 0.5 | </= 0.12 | >8 | 0.12 | </= 0.06 | >128 | - | - | blaCMY-42 | - |
| 129119 | EC | R | 0.25 | 4 | </= 0.25 | 8 | 2 | 0.5 | 1 | 0.25 | </= 0.25 | </= 0.06 | </= 0.06 | 64 | - | - | blaCMY-2 | - |
| 129181 | EC | B | 32 | >64 | >32 | >32 | >32 | >16 | >32 | 32 | >8 | >32 | >32 | >128 | blaNDM-5, blaOXA-181 | - | - | blaTEM-1B |
| 129201 | Eclo | B | 0.5 | >64 | 0.5 | 2 | 1 | >16 | 0.5 | 0.25 | >8 | </= 0.06 | </= 0.06 | 8 | - | blaCTX-M-15 | blaACT-5 | blaOXA-1, blaTEM-1B |
| 129439 | KP | R | 0.25 | 32 | 2 | >32 | >32 | >16 | 32 | 0.5 | >8 | 16 | </= 0.06 | >128 | blaKPC-2 | - | - | blaSHV-11, blaTEM-1B |
| 129456 | Eclo | B | 0.25 | 1 | 0.5 | 8 | 1 | 0.5 | 0.5 | 0.25 | </= 0.25 | </= 0.06 | </= 0.06 | 64 | - | - | blaACT-5 | - |
| 129470 | KA | B | 0.12 | 0.12 | 1 | 8 | 2 | </= 0.25 | 2 | 0.5 | </= 0.25 | 0.5 | </= 0.06 | 64 | - | - | ampC | - |
| 129502 | KP | W | 1 | >64 | 2 | >32 | >32 | 2 | >32 | 0.5 | >8 | >32 | 0.25 | >128 | blaKPC-3 | - | - | blaSHV-11 |
| 129647 | SM | R | 1 | 2 | 1 | 8 | 0.5 | 2 | 2 | 2 | </= 0.25 | 0.12 | 0.12 | >128 | - | - | blaSRT-2 | - |
| 129722 | KA | B | 0.12 | 0.25 | </= 0.25 | 4 | 1 | 0.5 | 1 | 0.25 | </= 0.25 | 0.12 | </= 0.06 | 8 | - | - | ampC | - |
| 129770 | Eclo | B | 0.25 | 1 | 0.5 | 4 | 1 | 0.5 | </= 0.25 | </= 0.12 | </= 0.25 | </= 0.06 | </= 0.06 | 32 | - | - | blaACT-16 | - |
| 129803 | Eclo | B | 0.12 | 0.5 | 1 | 8 | 1 | </= 0.25 | 1 | 0.25 | </= 0.25 | 0.25 | </= 0.06 | 32 | - | - | blaMIR-14 | - |
| 129832 | KP | R | 0.12 | 2 | </= 0.25 | 2 | 2 | >16 | 2 | 0.5 | 1 | 2 | </= 0.06 | 128 | blaKPC-2 | blaSHV-12 | - | blaTEM-1B |
| 129858 | Eclo | R | 0.06 | 1 | 1 | 4 | 1 | </= 0.25 | 0.5 | 0.25 | </= 0.25 | 0.12 | </= 0.06 | 32 | - | - | blaMIR-3 | - |
| 129901 | Eclo | R | 1 | 8 | 1 | 16 | 2 | </= 0.25 | </= 0.25 | </= 0.12 | 0.5 | 0.12 | </= 0.06 | >128 | - | - | blaACT-7 | - |
| 129936 | Eclo | R | 0.25 | 0.5 | 1 | 4 | 2 | 0.5 | 1 | 0.5 | </= 0.25 | 0.25 | </= 0.06 | 8 | - | - | blaACT-7 | - |
| 131259 | KA | B | 2 | 16 | 8 | >32 | >32 | 1 | >32 | 4 | 0.5 | 32 | 8 | >128 | - | - | ampC | - |
| 131416 | CF | R | 0.12 | 0.12 | </= 0.25 | </= 0.25 | 0.5 | </= 0.25 | 0.5 | 0.25 | </= 0.25 | </= 0.06 | </= 0.06 | 2 | - | - | blaCMY-65 | - |
| 131842 | EC | W | 1 | >64 | 1 | >32 | >32 | 1 | 2 | 0.25 | >8 | 8 | 1 | >128 | - | blaCTX-M-15 | - | - |
| 132161 | KO | R | 0.06 | 0.12 | </= 0.25 | 0.5 | 0.5 | 0.5 | </= 0.25 | 0.25 | </= 0.25 | </= 0.06 | </= 0.06 | 2 | - | - | blaPAO | blaOXA-50, blaOXY-2-5 |
| 132526 | Eclo | R | 0.12 | 0.12 | 0.5 | 1 | 0.5 | 0.5 | 0.5 | 0.25 | </= 0.25 | </= 0.06 | </= 0.06 | 2 | - | - | blaACT-7 | - |
| 133510 | KP | B | 0.5 | 32 | </= 0.25 | >32 | >32 | 1 | 16 | </= 0.12 | >8 | 32 | </= 0.06 | >128 | blaKPC-2 | blaSHV-12 | - | blaOXA-9 |

^a^ Organisms: CF, *Citrobacter freundii*; EC, *Escherichia coli*; EClo, *Enterobacter cloacae*; KA, *Klebsiella aerogenes*; KO, *Klebsiella oxytoca*; KP, *Klebsiella pneumoniae*; MM, *Morganella morganii*; PM, *Proteus mirabilis*; SM, *Serratia marcescens*.

^b^ Isolate sources: B, blood; R, respiratory; U, urine; W, wound.

^c^ Includes all other β-lactamases that are not carbapenemases, extended-spectrum β-lactamases or class C enzymes.

CP, carbapenemase; ESBL, extended-spectrum β-lactamase; FTB, cefepime/taniborbactam; CPM, cefepime; CZA, ceftazidime/avibactam; C/T, ceftolozane/tazobactam; ETP, ertapenem; GEN, gentamicin; IMI, imipenem; IMR, imipenem/relebactam; LEV, levofloxacin; MER, meropenem; MEV, meropenem/vaborbactam; PTZ, piperacillin/tazobactam.
